# Public Opinion and Cyberterrorism

**DOI:** 10.1093/poq/nfad006

**Published:** 2023-04-03

**Authors:** Ryan Shandler, Nadiya Kostyuk, Harry Oppenheimer

**Affiliations:** Postdoctoral Research Fellow, Blavatnik School of Government and Nuffield College, University of Oxford, Oxford, UK; Assistant Professor, School of Public Policy and the School of Cybersecurity and Privacy, Georgia Institute of Technology, Atlanta, GA, US; PhD candidate, Department of Government, Harvard University, Cambridge, MA, US

## Abstract

Research into cyber-conflict, public opinion, and international security is burgeoning, yet the field suffers from an absence of conceptual agreement about key terms. For instance, every time a cyberattack takes place, a public debate erupts as to whether it constitutes *cyberterrorism*. This debate bears significant consequences, seeing as the ascription of a “terrorism” label enables the application of heavy-handed counterterrorism powers and heightens the level of perceived threat among the public. In light of widespread conceptual disagreement in cyberspace, we assert that public opinion plays a heightened role in understanding the nature of cyber threats. We construct a typological framework to illuminate the attributes that drive the public classification of an attack as cyberterrorism, which we test through a ratings-based conjoint experiment in the United States, the United Kingdom, and Israel (N = 21,238 observations). We find that the public (1) refrains from labeling attacks by unknown actors or hacker collectives as cyberterrorism; and (2) classifies attacks that disseminate sensitive data as terrorism to a greater extent even than physically explosive attacks. Importantly, the uniform public perspectives across the three countries challenge a foundational tenet of public opinion and international relations scholarship that divided views among elites on foreign policy matters will be reflected by a divided public. This study concludes by providing a definitive conceptual baseline to support future research on the topic.

According to a 2021 Gallup study, the US public ranked “cyberterrorism” as the single most critical threat facing the nation—more even than Russian aggression, the spread of COVID, or the development of nuclear weapons by Iran (Brenan 2021). Some experts seem to share the public’s concern over a potent, even apocalyptic threat of cyberterrorism (Gross, Canetti, and Vashdi 2016, 2017; Henschke 2021), while others view this issue as a hyperbolic depiction of a still developing phenomenon (Valeriano and Maness 2015; Lawson 2019; Gomez and Whyte 2021). Yet what attacks are people afraid of? Which attacks fall under the heading of cyberterrorism, and do the public and elites share the same view of this phenomenon?

The dilemma of whether attacks constitute cyberterrorism is becoming increasingly common as cyberattacks proliferate. In the aftermath of the 2021 Colonial Pipeline cyberattack that triggered gas shortages throughout the United States, a vigorous public debate erupted as to whether this and similar attacks should be treated as terrorism. Some politicians excoriated the Biden administration’s characterization of the attack as merely criminal, declaring it a clear act of cyberterrorism that demands a commensurate response (Chamberlain 2021). Other public voices defended classifying the attack as cyber-crime, claiming that the facts do not warrant the application of a terrorist label, which should be reserved for a very specific subset of threats (Sanger and Perlroth 2021). This pattern recurs each time an attack is launched, with supporters pointing to the major consequences of the attack (Stroobants 2018), and detractors insisting on the absence of sufficient political intent (Rid 2013). The decision about whether to label a cyberattack as cyberterrorism bears significant implications, with the application of a terrorism label activating far-reaching counter-terrorism powers, and amplifying public fear and anger (Canetti-Nisim et al. 2009; Zeitzoff 2014; Snider et al. 2021).

In this article, we adopt experimental methods to explore the perception of cyberterrorism from the perspective of the wider public. While previous research has mapped the attitudes of the media (Zeri and Noordin 2017) and the positions of cybersecurity experts (Jarvis and Macdonald 2015), the views of the public remain understudied. This is surprising due to the central role that public opinion plays in understanding the effectiveness of terrorism (Enders and Sandler 2011). If a primary goal of terrorists is to spread fear and draw attention to their cause, then the public perception of what constitutes cyberterrorism is prerequisite to gauging its effects. In addition to entering this theoretical debate, this manuscript aims to generate a level of conceptual consistency to guide future research. Empirical and theoretical research into cyber-conflict, public opinion, and international security is burgeoning, yet the field suffers from an absence of conceptual agreement about key terms (Branch 2021). This article seeks to provide a definitive baseline to support the wave of research on the topic.

To probe the factors that underlie the decision to designate a cyberattack as cyberterrorism, we employ a ratings-based conjoint experimental design with 3,036 respondents in three countries—the United States, United Kingdom, and Israel. We begin by constructing a typology of key variables that we hypothesize will drive the public perception of cyberterrorism. We then employ a conjoint structure to isolate the effect of each attribute on the decision to classify various scenarios as cyberterrorism. Building on this base, we take advantage of our multi-country dataset to examine whether the attributes that drive the designation of cyberterrorism are uniform across borders. This design sets our paper apart as the most comprehensive comparative study of public opinion in response to cyberattacks.

We identify several definitive features that guide the public classification of cyber incidents. First, we find that the public is disinclined to label attacks by unknown actors as cyberterrorism, which is striking due to the difficulty of attributing cyberattacks. Second, we observe a clear reticence to associate attacks by individuals or hacker collectives as cyberterrorism, regardless of the consequences of such attacks. Third, we detect a readiness to associate attacks that steal and disseminate sensitive data with cyberterrorism, to the extent that the public views these attacks just as seriously as attacks causing major physical explosions. Overall, our results demonstrate a highly stable view of cyberterrorism, which transcends national boundaries, and which starkly differs from elite-driven understandings of terrorism. Given the absence of conceptual uniformity among domain experts (Jarvis and Macdonald 2015), we conclude by arguing that the public perspective on cyberterrorism may constrain policy elites due to the robust public consensus that has formed.

## Why Is Public Opinion Important in Understanding Cyberterrorism?

Historically, the public was viewed as holding inconsistent and incoherent views on national security and foreign policy, which led to policymakers ignoring their views and limited their influence over policy (Almond 1950; Baum and Potter 2015). However, scholars have come to understand that the public has increasingly nuanced views on matters of national security, and that public opinion can influence elite decision-making (Lin-Greenberg 2021; Sevenans 2021). While we do not go so far as to claim that public views can definitively resolve technical policy debates, we offer four reasons why civilian responsiveness to cyberattacks is important.

First, the public perception of an attack as terrorism has political and electoral consequences. Leaders frequently take advantage of the ambiguity inherent in terrorism to designate the acts of out-group members as terrorism and reap political benefits (Geis and Wunderlich 2014). Similarly, the public often demands that politicians label acts of domestic violence as terrorism due to the normative weight of the label. A seminal example of this is the acrimonious public debate in the United States about applying the terrorist tag to acts by white-nationalist groups (Ackerman 2012). In addition to the normative significance of this labeling act, designating an out-group as terrorists strongly impacts voting behavior and election outcomes (Montalvo 2011). The extent to which the public will accept or expect such designations in the realm of cyber is still open to question.

Second, the subjective perception of an act as terrorism can influence political attitudes ranging from support for military action to shifts in political orientation (Getmansky and Zeitzoff 2014). These political effects are mediated by the subjective perception of threat (Huddy et al. 2005; Snider et al. 2022). For instance, perceiving an attack as terrorism rather than crime leads to distinct levels of anxiety and fear (Shechory-Bitton and Cohen-Louck 2018), which in turn shifts political attitudes such as militaristic tendencies, and willingness to sacrifice civil liberties for security (Snider et al. 2021). Applying this to cyberspace, it may well be that the public perception of an attack as cyberterrorism rather than cyber-crime or cyber-vandalism will likewise bear an outsize effect on political behavior.

Third, the media regularly has to make decisions about how to frame acts of political violence. This often takes place before the authorities have made a public determination about whether an act formally meets the designation of terrorism. With the diffusion of media from a centralized elite to independent reporters, bloggers, and influencers operating through social media, this framing decision is moving from professional editors to individual actors (Konow-Lund and Olsson 2017). Seeing as the framing of an act as terrorism can significantly raise the profile of an attack and influence the public’s response (Baum and Groeling 2010), it becomes important to understand the factors that drive journalists and thought leaders to designate an attack as terrorism.

Finally, there is often greater room for public input in policy areas lacking broad consensus, since elites are more willing to follow popular views in these cases (Kreps and Das 2017). This situation can be aptly observed in the case of cyberterrorism, where elites have demonstrated that they are attuned to public views regarding cyberterrorism. For instance, Congress members in the United States and parliamentarians in the United Kingdom have made speeches on the floors of their respective houses that specifically referred to the views of their constituents about the nature and gravity of the cyberterrorism threat (U.S. Congress 2016).

In summary, we assert that the public is a central actor in shaping the normative and political significance of cyberterrorism. It is well established that public opinion plays a tangible albeit limited role when it comes to traditional foreign policy issues, and we expect that this role will be greater still when it comes to novel cyber-threats where the absence of established norms and the absence of an elite consensus on the definition of cyberterrorism open the door to greater public influence.

## When Do Cyberattacks Become Cyberterrorism in the Eyes of the Public?

A number of studies over the years have sought to define cyberterrorism, yet what emerges chiefly from these analyses is the complete lack of consensus about the term (Luiijf 2014). Jarvis, Nouri, and Whiting (2014) explain the contestability of this phenomenon by pointing to the still-evolving nature of cyberspace and the collective fear of omniscient attackers whose destructive power is inflated in the public discourse. In the absence of consistent policy guidance, which attributes does the public rely on in classifying a cyberattack as cyberterrorism?

Insofar as cyberterrorism is just a methodological subset of terrorism, the public may search for characteristics it associates with conventional terrorism. Yet while cyberterrorism is unquestionably related, research has shown that it is substantively different from conventional terrorism in its practice and effects (Backhaus et al. 2020; Shandler, Snider, and Canetti 2022; Shandler and Gomez 2022; Shandler et al., 2023). We therefore hypothesize that there will be several distinguishing predictive features that are unique to cyberterrorism.

To identify these features, we draw on an enduring debate in the literature about whether the focal cyber component of cyberterrorism refers to the ends or means of an attack. Specifically, Denning (2000) suggested that cyberterrorism should be generally understood to mean unlawful attacks and threats thereof against computer networks. This view of cyberterrorism gained traction with supporters who extrapolated that cyberterrorism refers to digital political acts as varied as unauthorized deletions and denial-of-service attacks (Luiijf 2014). These scholars stress the importance of a digital *target* for an act to be designated as cyberterrorism. By contrast, most researchers today subscribe to a broader conception of the term that views any attack predominantly utilizing digital means as meeting the cyber-threshold of cyberterrorism, even if the final target is not digital (Murray et al. 2019). For these scholars, a digital *method* is a priority.

In addition to the “target” and “method” classes, we also distinguish three additional categories—outcome, agent, and motivation—which form cues that help people classify attacks. We explain each of the five categories below and how they contribute to the public ascription of cyberterrorism. We end this section by hypothesizing about the cross-national peculiarities that might contribute to diverging views of cyberterrorism among countries.

### Factors Guiding the Public Ascription of Cyberterrorism

#### Method of attack

The form of the perpetrated violence is a central variable in attributing an attack as terrorism. Referring to our definitional dilemma, we first distinguish whether an attacker uses digital or non-digital means to execute an attack. Following Murray et al. (2019), we hypothesize that an attack utilizing digital means should increase the public perception of an attack as cyberterrorism more than an attack utilizing non-digital means. Yet we also differentiate between different types of digital attacks. Studies of conventional terrorism show that the method of attack matters, with bombings perceived as most highly associated with terrorism, followed by less destructive methods such as shootings and stabbings (Huff and Kertzer 2018). Even though we substitute these kinetic methods with digital equivalents such as malware, computer viruses, and Trojan horses, we expect that the particular digital method of attack will not be predictive of an attack’s classification as an instance of cyberterrorism, since no one attack type is considered more dangerous or destructive than another.

#### Target of attack

We differentiate between the targets of an attack in two ways. First, relating to abovementioned definitional dilemma, we distinguish whether the digital element of cyberterrorism must be the means of attack or the target of attack. Following Denning (2000), we hypothesize that digital targets (i.e., network servers) should increase the public ascription of cyberterrorism more than non-digital targets. Second, and following the literature on conventional terrorism, we distinguish between military and noncombatant targets. The public view of terrorism typically accords with the jus in bello perspective that recognizes the legitimacy of attacks on combatants, and only views attacks against noncombatants as terrorism. Yet the advent of cyber-capabilities has complicated this balancing act, since cyber-operations can deliver major advantages without causing fatalities, a fact that should minimize the stigma against targeting noncombatants (Gross 2018).

#### Outcome of attack

The question of whether cyberattacks need to cause real-world physical destruction is central to our debate on perceptions of cyberterrorism. Previous research has shown that cyber-terror acts must result in physical destruction to trigger strong public support for military retaliation (Shandler, Gross, and Canetti 2021; Shandler et al. 2022). As such, we expect that the “outcome of attack” attribute will be significant in predicting whether the public ascribes a terrorism label to a cyberattack—attacks that cause physical destruction will be classified as terrorism, while those with an outcome of financial theft will not.

#### Agent of attack/attacker identity

Most definitions of terrorism require that the perpetrator be either conducted or inspired by an organization with an identifiable chain of command (Hoffman 2006). Previous research suggests that the public is more likely to consider an organizationally linked attack as terrorism compared to attacks by individuals (Huff and Kertzer 2018). Cyber-attackers can be divided in the same way, and so we expect that the involvement of organized groups will be predictive of a cyberterrorism label, compared with individual hackers (D’Orazio and Salehyan 2018). But, in contrast to kinetic attacks, the identities of cyber-attackers often remain unknown since the particular characteristics of cyberspace allow actors to obfuscate their identity (Rid and Buchanan 2015). The literature is divided on the effects of this uncertainty in attribution on public attitudes. Drawing on theories of risk perception, several scholars have theorized that the view of digital actors as omniscient, invisible perpetrators will heighten threat perception (Jarvis, Nouri, and Whiting 2014; Dunn Cavelty 2019; Kostyuk and Wayne 2021). But recent research suggests that the absence of definitive attribution will decrease the likelihood that the attack will be perceived as terrorism (Bada and Nurse 2020). In line with the latter logic, we expect that the public will view attacks with unattributed perpetrators as less likely to be a cyberterrorism event.

#### Motivation of attack

The motivation attribute can be considered as a subjective determination since it requires the public to explore the underlying intent of another person. Yet the public intuitively relies on assumptions about attacker motivation in forming opinions (Kimhi, Canetti-Nisim, and Hirschberger 2009). Therefore, we argue that the public will take note of the intent behind an attack as a factor in how the attack is perceived (Canetti, Gubler, and Zeitzoff 2021). Common motivations associated with terrorism include political, social, and religiously driven attempts to influence public policy, though states have begun to broaden the definition of terrorism with new laws that equate individual hate crimes with domestic terrorism. Furthermore, we note that in contrast to conventional terror attacks, the motivation of cyberattackers often remains unknown (Rid and Buchanan 2015). In line with this logic, we expect that the public will view attacks with unknown motivations as less likely to be a cyberterrorism event.

#### Country-level effects

To what extent will public views transcend national borders? We suggest that the above attributes will regulate the public perception of cyberterrorism across countries. The reason for this is that the public is exposed to extensive discourse on cyber-threats via the media and popular culture—which tends to be homogeneous in its depictions of cyberterrorism (Zeri and Noordin 2017).^1^ To the extent that the media adopts a consistent approach to reporting about cyberterrorism, then any elite variance will not necessarily filter down to the public (Groeling and Baum 2008; Kertzer and Zeitzoff 2017). If this is indeed the case, we should see a consistent view of cyberterrorism within and among the three countries in our research sample—the United States, United Kingdom, and Israel.

Nevertheless, despite our expectation of a consistent perception of cyberterrorism, we do expect that there will be some level of country-level divergence based on the distinctive experiences of particular countries. For example, distinct to the United States is the politicization of database breaches by individual hackers. Seminal examples include the Snowden leaks, and the dissemination of troves of emails from the Democratic National Committee during the 2016 national election. The highly public response to these hacks has led to a charged public debate about information theft, cyberattacks, and treason. We therefore expect that American respondents will perceive cyberattacks by individual hackers as cyberterrorism to a greater extent than other countries. Likewise, we expect a Jihadi attacker identity to be significantly predictive of the application of a cyberterrorism label in all countries (D’Orazio and Salehyan 2018), yet we anticipate that this will be strongest in Israel, where religious violence is most salient.

## Method

**Figure 1.**
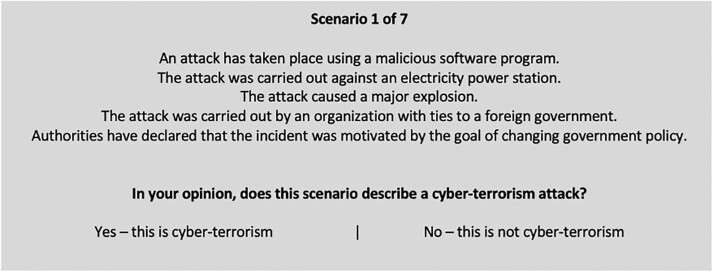
An example conjoint scenario.

Our analytical strategy involves estimating the causal effects of specific incident attributes on the public ascription of an attack as cyberterrorism. To do so, we use a conjoint experiment design that works by building randomly generated scenarios with interchangeable attributes that are drawn from a pre-populated list. After viewing a randomly generated scenario, populated with one item drawn from each of the five incident attributes explained in our theory section, participants responded whether they would or would not classify the incident as cyberterrorism. We use a conjoint design because it: (1) allows us to hold fixed a multitude of attributes—something that would not be possible with observational studies due to the inability to isolate the variables of interest, and (2) gives us the ability to study the effect of each attribute, which would not be possible with traditional factorial experiments due to the statistical power constraints. We consider a ratings-based conjoint better suited to this study than a choice-conjoint structure since citizens assess the status of each attack as they appear, rather than comparing the merits of two attacks against one another. Figure 1 illustrates how a randomly generated vignette appeared to participants.

### Survey Instrument

In this section, we explain the five-attribute typology which we employ in our conjoint design.^2^Table 1 summarizes the conjoint attributes, values, and reference conditions.

**Table 1.** Attributes for cyberterrorism classification scenarios in conjoint experiment.

Attributes	Values
Agent of attack	A foreign country (baseline)
	A group of hackers with ties to a foreign government
	A Jihadi Islamic group
	An organization with ties to a foreign government
	An unaffiliated group of hackers
	An individual hacker
	An unknown actor
Method of attack	A cyberattack has taken place (baseline)
	An attack has taken place
	An attack has taken place through the insertion of an infected USB disc
	An attack has taken place using an email that tricked employees into sharing their passwords and usernames
	An attack has taken place using a malicious software program
Motivation of attack	The incident was motivated by revenge (baseline)
	The incident was motivated by the goal of overthrowing the government
	The incident was motivated by the goal of changing government policy
	The motivation for the incident remains unknown
Outcome of attack	No casualties or property damage were reported in the attack (baseline)
	The attack caused a major explosion
	Sensitive personal and organizational data was stolen and publicly released in the attack
	The attack caused a minor explosion
	Millions of dollars were stolen in the attack
Target of attack	A military facility (baseline)
	Online databases (containing personal information about citizens)
	A government office building
	An electricity power station
	A shopping mall

*Note:* This table shows the attributes and attribute values that are used to generate the cyberterrorism scenarios for the conjoint methodology. For the purposes of statistical comparisons, one value in each attribute category is marked as the “baseline item” against which all of the other items are measured.

#### Attribute #1: agent of attack

Following the literature on conventional terrorism, we distinguish between terror organizations (independent versus state-sponsored) and lone-wolf attacks (Weimann 2012). We also add a new digital-centric category of “loosely affiliated hacker group” in recognition of the prominent hacktivist groups that are responsible for widely reported cyberattacks and which do not fit into classical actor categories. Finally, since many cyberattacks lack attribution, we label them as an “unknown actor.” We use a “foreign country” as our baseline.^3^

#### Attribute #2: method of attack

The method of attack attribute distinguishes between purely external intrusions such as malware, spear-phishing attacks, and insider-facilitated intrusions such as an infected USB drive plugged into a target site. We included the above categories because they constitute common forms of cyberattacks that are often discussed in the public sphere. A generic unspecified “cyberattack” was inserted as a baseline condition.

#### Attribute #3: motivation of attack

The public often takes note of the intent behind an attack as a factor in how the attack is perceived (Canetti, Gubler, and Zeitzoff 2021). Potential motivations included in our typology range from an explicit desire to overthrow the government, to a somewhat softer intent to alter government policy, or to a simple revenge motive, which we use as our reference condition. Since many cyberattacks lack attribution, we also include an unknown motivation behind an attack.

#### Attribute #4: outcome of attack

The existence and the extent of casualties in the aftermath of attacks attract significant public attention. While this outcome variable is usually measured on a continuum of no casualties to mass casualties, we intend to consider several other nonlethal outcomes that relate to the particular nature of cyberterrorism. These include the theft of sensitive personal or organizational data, and financial theft. A baseline option was included whereby the attack caused no casualties or property damage.

#### Attribute #5: target of attack

We differentiate between the targets of an attack in two ways. First, to consider whether the digital element of cyberterrorism must be the means of attack or the target of attack, we include both digital targets (e.g., databases) and traditional concrete targets (e.g., malls or power stations). Second, we distinguish between military (e.g., a military facility) and noncombatant (e.g., a shopping mall) targets. Similarly, we employ this distinction because each of these types of targets are frequently the subject of cyberattacks (e.g., Wheeler and Schneier 2021). Military targets are used as a baseline condition.

Adopting best practices in conjoint experiments in the political sciences (see Hainmueller and Hopkins 2015), we minimize the number of design constraints, adding only two constraints within the randomization protocols to avoid implausible combinations of scenario attributes. Specifically, we excluded scenarios depicting an attack on information databases causing a major or a minor explosion. In total, there are 3,220 unique ways to build a scenario by substituting the different values in each attribute.

### Survey Sample and Design

The study was fielded simultaneously in the three countries on 26 August 2020, during which time we recruited 3,242 respondents (United States, N = 1,077; United Kingdom, N = 1,042; Israel, N = 1,123). Surveys were distributed via Amazon Mechanical Turk, Prolific, and the Midgam Survey company in the United States, the United Kingdom, and Israel, respectively. Participants were required to have an approval rating of 96 percent, and reCAPTCHA technology was used to weed out potential bots. We retain only the 3,036 respondents who completed all of the conjoint questions and who passed the reCAPTCHA test. This amounts to a total non-completion rate of 6.35 percent—a rate that compares favorably to completion metrics in other web-based surveys (Liu and Wronski 2018). Following best practice, we refrain from removing speeders (Greszki, Meyer, and Schoen 2015).^4^

We elect to focus on three countries to demonstrate that our hypothesized effects transcend borders. Even though cultural differences often precipitate distinct national responses to security crises, we hypothesized that public opinion regarding cyber threats should be stable cross-nationally. We focus on these three countries since they possess certain features in common. Importantly, they are all technologically advanced countries with a history of publicly reported cyberattacks on critical infrastructure (Critifence 2018). Additionally, they are all democracies, where public opinion can have a greater effect on policy. In the Discussion section, we consider the generalizability of these scope conditions. With 3,036 participants classifying seven incidents each, the following analyses include classifications of 21,238 different randomly generated scenarios.

The following analyses are carried out using the R Statistical Computing language (v4.2.1, R Core Team 2022), and RStudio Version 1.2.1335. The conjoint analyses were carried out using a combination of functions from the *Cregg* and *Cjoint* packages, respectively (Barari et al. 2018; Leeper 2020). We cluster all standard errors by respondent, seeing as rating outcomes are not independent across the multiple scenarios rated by each respondent. A codebook with details of all collected variables appears in Supplementary Material section 1.

## Results

We begin by analyzing which attack attributes heighten the likelihood of a cyber incident being perceived as cyberterrorism. We then proceed to disaggregate the results across countries.

### Effects of Attack Attributes on Classification of Incidents as Cyberterrorism

**Figure 2.**
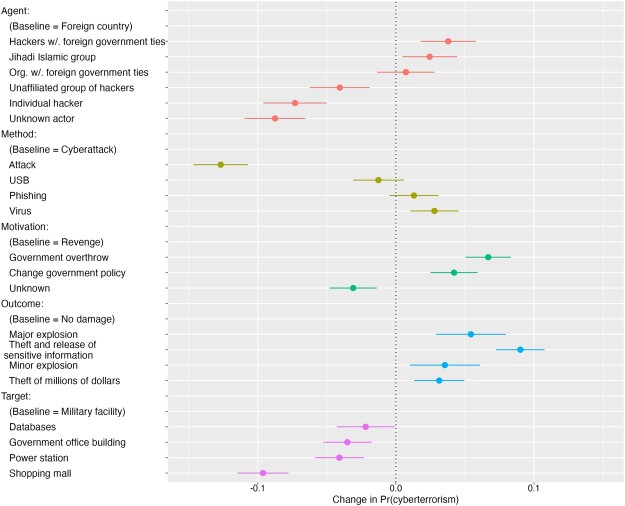
Effects of incident attributes on probability of being classified as cyberterrorism. This plot depicts estimates of the effects of each randomly assigned attribute values on the probability of a scenario being classified as cyberterrorism. Estimates are based on the AMCE model with respondent-clustered standard errors. Bars represent 95 percent confidence intervals. This plot reveals a pooled estimate for the full multicountry sample (N = 21,238 observations from 3,036 unique respondents).

Our analysis strategy involves assessing the relative importance of each attribute by calculating the average marginal component effects (AMCE). This draws on an estimation strategy developed by Hainmueller, Hopkins, and Yamamoto (2014), which has since become the norm in conjoint analyses in the political sciences (Hainmueller and Hopkins 2015).^5^ The AMCE figure quantifies the differential likelihood of a scenario being classified as cyberterrorism when comparing two attribute values while controlling for all other permutations of the remaining attributes. Figure 2 displays the results for each of the five attributes among the full multicountry sample.^6^ The lines indicate 95 percent confidence intervals for the AMCE of each attribute value—reflecting the change in the probability that respondents will classify a scenario containing that value as cyberterrorism. The upper items in each category denote the reference item for that attribute against which the other values are compared.

A number of trends immediately emerge from figure 2. Taking note of the effect of the *attack agent*, we can observe a clear reticence to associate non-governmental actors with cyberterrorism. Compared to the baseline (foreign country), the presence of an individual hacker or an unaffiliated group of hackers significantly decreased the likelihood that an attack would be classified as cyberterrorism by 7.3 percentage points (SE = 1.2) and 4.1 percentage points (SE = 1.1), respectively. This means that, all else being equal, the wider public is likely to eschew the label of cyberterrorism when assessing the actions of an unaffiliated hacker collective like Anonymous. By contrast, the public appears to be willing to make accusations on the basis of circumstantial evidence, and will label attacks as cyberterrorism if ostensibly independent hackers maintain ties with a foreign government. This reinforces new research by Canfil (2022) that overturned the fallacy of a widespread and successful use of non-state proxies to obscure attribution in cyberspace. Notably, the presence of an unknown actor significantly diminishes the likelihood that an incident will be labeled as cyberterrorism in the eyes of the public (8.7 percentage points; SE = 1.1).

Turning to the *method of attack*, we can immediately see a clear division between non-cyber-specific items (an attack), and attack descriptors that convey that a cyber-technique was employed (phishing attack, virus, etc.). Describing the methodology of an incident as “an attack” significantly lowers the likelihood of an incident being designated as cyberterrorism by 12.7 percentage points (SE = 1.0) compared to a “cyberattack” description. What is noteworthy here is that there is conspicuously little difference between the various cyber methodologies. Even a generic “cyberattack” is sufficient to invoke the cyber connection that the public ostensibly seeks. This tells us that the public does not differentiate between alternate cyberattack routes when classifying an incident. The public responds in the same way whether an attack utilizes a phishing technique or an infected USB key.

We also find evidence that *attacker motivation* matters, with actions spurred by a desire to change government policy or overthrow the government leading, naturally, to a significantly higher probability of incidents being classified as cyberterrorism compared to revenge as the motivation (4.2 percentage points; SE = 0.9 and 6.7 percentage points; SE = 0.8). This abides by the same internal logic that drives the definition of conventional terrorism, requiring some underlying political motive. What stands out is the contractionary effect of the perpetrator’s motivation being unknown. Similarly to the presence of an unknown attacker, the lack of specificity in an attacker’s motivation significantly reduces the perception of an attack as constituting cyberterrorism (3.1 percentage points; SE = 0.9) when compared to a vengeance motivation, which is not typically associated with terrorism.

Looking at the *outcome of an attack*, we can see that attacks causing minor or major physical destruction will significantly increase the likelihood of an incident being designated as cyberterrorism compared to an attack that leads to no damage by 3.5 and 5.4 percentage points, respectively. This confirms prior research by Shandler et al. (2022), which emphasized the importance of tangible physical effects in shifting public opinion following strikes in the cyber domain. Yet our findings reveal that the public views the theft and dissemination of sensitive personal and organization data as an even graver consequence, significantly increasing the likelihood of a cyberterrorism classification by 9.0 percentage points (SE = 0.9) compared to the baseline. This may reflect a mounting comprehension of the damage that can be wreaked by the dissemination of personal data, and the continual exposure to reports of foreign actors targeting sensitive data in the context of national elections (Zubiaga, Procter, and Maple 2018). By contrast, an outcome of monetary theft is not nearly as likely to invoke the specter of cyberterrorism compared to major physical destruction and data acquisition.

Finally, the *attack target* attribute reveals the limits of the public’s comprehension of terrorism, with military facilities being most highly associated with cyberterrorism, more than government office buildings, power stations, or shopping malls. One of the rare features that is shared among all definitions of terrorism and cyberterrorism is that it must target civilians—not combatants. As such, it is surprising to see that attacks against civilian infrastructure such as shopping malls significantly lowered the likelihood that an incident would be classified as cyberterrorism by some 9.6 percentage points (SE = 0.9) compared to an attack on a military facility. A possible explanation is that the vast majority of high-profile cyberattacks that have been reported in the media have been attacks against the state itself (Jarvis, Macdonald, and Whiting 2017). By contrast, reports of cyberterrorism attacks targeting civilian infrastructure have only begun to emerge in the last three years (Sanger and Perlroth 2021), and so the public may simply not have paradigmatic examples of cyberterrorism targeting civilians in mind. Alternatively, Young and Findley (2011) have raised the possibility that attacks against military targets—conducted during peacetime—might reasonably qualify for the category of terrorism. For example, the bombing of the USS *Cole* in 2000, and the September 11 plane attack on the Pentagon, are both commonly referred to as terrorism, even though the attacks were conducted against military targets. In our conjoint study, the participants received no hint that any attack took place in the context of active military operations, and so it is natural that they would view unprovoked attacks on the military as terrorism.

### Comparing Cyberterrorism Classifications across Country Samples

**Figure 3.**
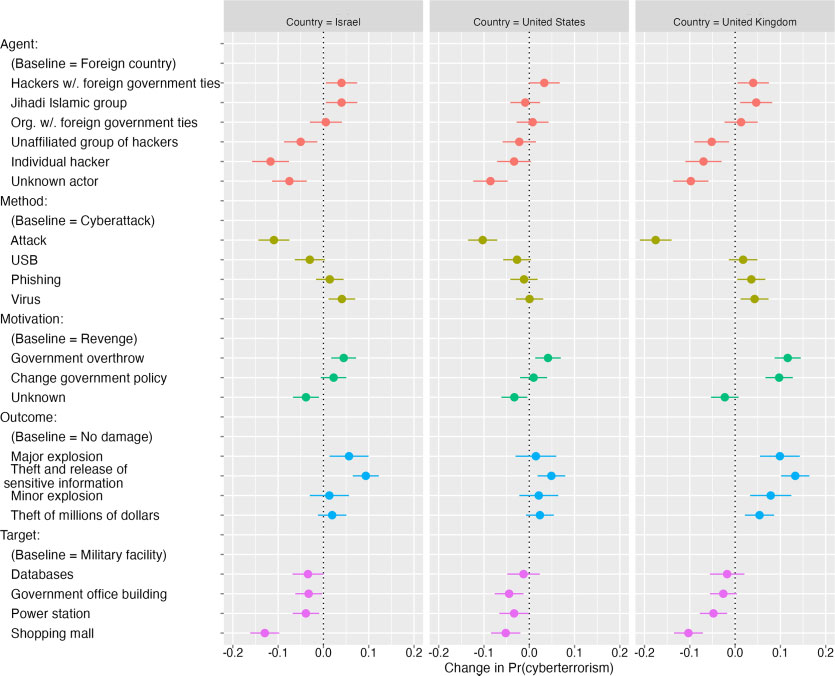
Effects of incident attributes on probability of being classified as cyberterrorism—by country. This plot depicts estimates of the effects of each randomly assigned attribute value on the probability of a scenario being classified as cyberterrorism. Estimates are based on the AMCE model with clustered standard errors. Bars represent 95 percent confidence intervals. This plot exhibits a pooled estimate for each country sample (N = 7,084 observations from 1,012 respondents in the United States; 7,070 observations from 1,010 respondents in the United Kingdom; and 7,098 observations from 1,014 respondents in Israel).

In figure 3, we display the results for each of the five attributes again, but this time broken down by country. Each line indicates the 95 percent confidence interval for the AMCE of an attribute value—reflecting the relative probability that respondents will classify a scenario containing that value as cyberterrorism.^7^ It is immediately apparent that there is a high level of consistency between countries in how each attribute affects the classification of an incident as cyberterrorism.

**Figure 4.**
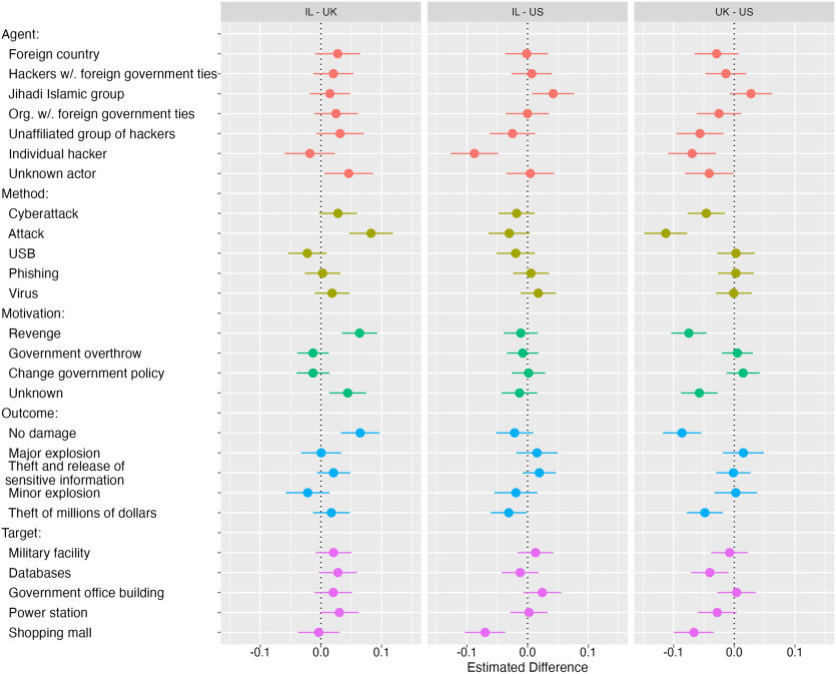
Marginal means differences by country. This figure contains a plot of the difference in marginal mean levels cyberterrorism attribution by treatment category with 95 percent confidence intervals. The country that appears first in the title is the reference condition, against which the country listed second is compared. For example, attributing the motivation of an attack to revenge has a more positive effect on attribution for American respondents than Israeli or British respondents. IL: Israel; UK: United Kingdom; US: United States.

To identify the extent of any between-country differences, we utilize a technique developed by Leeper, Hobolt, and Tilley (2020) that examines subgroup differences using conditional marginal means by highlighting the similarities and dissimilarities between subgroups without the distorting effect of a reference condition. This dyadic analysis appears in figure 4, and the full cross-country estimates appear in Supplementary Material section 3.

The key result to emerge from this analysis is that there is an extremely high degree of similarity across countries in how each attribute affects the public designation of an incident as cyberterrorism. Comparing between the United States and Israel, for example, there are only 4 of 26 attribute values that are statistically different at the 5 percent-level across the samples. It appears that despite the absence of clear top-down guidance, the publics in multiple countries have coalesced in their views of what constitutes cyberterrorism.

While the estimates across countries reveal a high degree of similarity, there are several isolated attributes where the effects do vary between countries. For instance, as we hypothesized, the US respondents were significantly less likely to label an attack by an individual hacker as cyberterrorism, compared to the United Kingdom (*p* < .001) and Israel (*p* < .001). British respondents were slightly more attuned to the motivation of the attackers, with revenge motivations and unknown motivations both significantly lowering the chances of an attack being classified as cyberterrorism compared to American (*p* < .001 for revenge and *p* < .001 for unknown motivation) and Israeli (*p* < .001 for revenge and *p* < .005 for unknown motivation) respondents.^8^

## Discussion

This study stems from the premise that there is substantial disagreement over what constitutes various cyber-threats. We witness this confusion in the aftermath of each cyber incident, with public debate flaring over whether to label the incident as cyberterrorism, cybercrime, cyber-vandalism, or something else entirely. We elected to focus on the wider public since the public designation of an act as terrorism has broad political consequences. If an objectively criminal attack is perceived as cyberterrorism by the general public, then this can heighten public fear and distort public confidence in law enforcement. By contrast, if a cyberattack by a terrorist organization is not perceived as cyberterrorism, then this minimizes the effectiveness of an attack.

Using a rating-based conjoint experimental mechanism, we isolated the extent to which specific incident attributes contribute to the ascription of an attack as cyberterrorism. We take note of several key findings that emerged from the study. Collectively, these results can settle much of the ingrained ambiguity about public opinion and cyberterrorism, and serve as the basis for future research.

Specifically, we observe a clear reticence to associate attacks by non-institutional actors with cyberterrorism. Though it resembles a semantic truism, cyberterrorism is waged by terrorist organizations in the eyes of the public, or at least by a hierarchical organization with ties to a foreign power. By contrast, attacks by individual hackers or loose collectives of hackers do not resemble terrorist attacks. This finding is likely to tamp down the prevalence of the cyberterrorism label, since traditional terrorist organizations have been slow to adopt cyberspace as an attack platform (Gartzke 2013).

Next, we note that even more than for individual hackers, the public is wary of labeling attacks by unknown actors as cyberterrorism. This circumspection is further amplified when the motivation of the attack is unknown. While it was once theorized that seemingly omniscient cyber-perpetrators would terrorize a hapless public, it is now accepted that the absence of any definitive attribution is likely to have the opposite effect, seeing as terrorists crave attention compared to mere cybercriminals that tend to operate in the shadows (Bada and Nurse 2020). This finding offers guidance for governments that increasingly view the public attribution of cyber perpetrators as a strategic decision—and who can “name and shame” an offender to heighten the likelihood that the public will view the attack as terrorism (Egloff 2020).

Our findings partially corroborate past research that identified that only cyberattacks causing physically destructive outcomes would rise to the level of cyberterrorism in terms of the political effects it levies on the public (Shandler et al. 2022). While destructive outcomes substantially increased the likelihood of an attack being viewed as cyberterrorism, surprisingly, the public viewed the dissemination of sensitive digital data as an even graver consequence, and freely attached the label of cyberterrorism to attacks of this sort. This reinforces recent findings that the public views attacks that disseminate sensitive data as seriously as physically destructive attacks (Zubiaga, Procter, and Maple 2018).

This study also revealed that no single cyber instrument (i.e., malware attacks, phishing attacks, etc.) is more reflective of cyberterrorism than any other. Even reports of a generic “cyberattack” are sufficient to meet the minimum threshold required to raise the specter of cyberterrorism. This finding is a key point of departure from public attitudes to conventional terrorism, where there is an innate ranking of terroristic methods (i.e., bombings imply terrorism, stabbings do not) (Huff and Kertzer 2018).

Finally, our findings speak more broadly to the public opinion literature relating to the overlap between elite guidance and public opinion pertaining to international security (Kertzer and Zeitzoff 2017). A tenet of public opinion and international relations scholarship holds that the public follows elite opinion in developing views on foreign policy and national security issues. We demonstrate that views on cyber issues, where elite guidance is divided, diverge from this classical model. Somehow, despite elite divisions, the public has formed a cohesive cross-national view of cyberterrorism. This finding bears important policy implications—since the uniform public perspective on cyber terrorism may constrain policy elites at the national and international levels in formulating policy due to the robust public norms that have already formed in this area.

Is it pertinent that media coverage of an attack may oftentimes fail to accurately depict the objective facts of an incident? We remain agnostic about this question given that our treatments reflect real-world reports of the kind that readers must frequently evaluate. Since the credibility of an information source can influence decision-making processes, future research would do well to explore the effect of source trustworthiness upon the ascription of an attack as cyberterrorism.

Our multicountry empirical sweep gives us confidence that our findings are not the product of any one mass public, and that the results are robust to national idiosyncrasies. We acknowledge that our empirics focus heavily on Western, democratic, and technologically advanced countries. While this offers a broad set of scope conditions, we must be wary of overbroad generalizations. We conclude by noting that we have identified a highly stable view of cyberterrorism among our cross-national sample, and this perspective diverges from the objective and elite-driven definition of terrorism. As such, this paper reinforces the emergence of a new relationship between public opinion and security issues when it comes to cybersecurity. As cyber-threats become ever more salient, this cleavage is poised to elevate the role of public opinion.

## Supplementary Material

nfad006_Supplementary_Data

## Data Availability

Replication data and documentation are available at https://doi.org/10.7910/DVN/FU62PS.

## Footnotes

1 While Jarvis, Macdonald, and Whiting (2017) identify diversity in media discourse, they confirm that the most apprehensive international media accounts of cyberterrorism are presented in a uniform manner as cataclysmic threats targeting “the West” or the entire world.

2 The research design and all hypotheses were preregistered. The pre-analysis plan can be accessed at https://osf.io/d69k3.

3 As a rule, neutral or status-quo items are selected as the baseline conditions against which the effect of substituting for other items are contrasted. In a subsequent analysis, we employ a marginal means technique which avoids the distorting effect of a reference condition (Leeper, Hobolt, and Tilley 2020).

4 Detailed balance checks and an extended discussion of the recruitment strategy and sample quality appear in Supplementary Material section 2.

5 We discuss whether and how our design abides by the assumptions required to conduct ratings-based conjoint analysis in Supplementary Material section 5.

6 The full coefficient table for Figure 2 appears in Appendix A.

7 The full tabular results for this analysis are in Appendix B.

8 In addition to examining country effects, we also measure the interaction between respondents’ partisan beliefs and computer literacy (Supplementary Material section 4). We find that: (1) liberals rely more on the presence or absence of particular features in applying a terroristic label; and (2) highly digital literate respondents are more attuned to the gravity of particular types of attack.

## Appendixes

### Appendix A. Pooled Results

Table A1 contains the full AMCE results for the pooled sample analysis that appears in figure 2.

**Table A1.** Average marginal component effect for pooled sample.

Attribute	Level	Estimate	Std. err	z value	*p*
Agent	Hackers with foreign government ties	0.038	0.010	3.731	.000
Agent	Jihadi Islamic group	0.024	0.010	2.420	.016
Agent	Organization with foreign government ties	0.007	0.011	0.684	.494
Agent	Unaffiliated group of hackers	−0.041	0.011	−3.680	.000
Agent	Individual hacker	−0.073	0.012	−6.257	.000
Agent	Unknown actor	−0.087	0.011	−7.725	.000
Method	Attack	−0.127	0.010	−12.627	.000
Method	USB	−0.013	0.009	−1.347	.178
Method	Phishing	0.013	0.009	1.447	.148
Method	Virus	0.028	0.009	3.141	.002
Motivation	Government overthrow	0.067	0.008	8.015	.000
Motivation	Change government policy	0.042	0.009	4.839	.000
Motivation	Unknown	−0.031	0.009	−3.569	.000
Outcome	Major explosion	0.054	0.013	4.204	.000
Outcome	Theft and release of sensitive information	0.090	0.009	10.106	.000
Outcome	Minor explosion	0.035	0.013	2.743	.006
Outcome	Theft of millions of dollars	0.031	0.009	3.372	.001
Target	Databases	−0.022	0.011	−2.056	.040
Target	Government office building	−0.035	0.009	−3.945	.000
Target	Power station	−0.041	0.009	−4.528	.000
Target	Shopping mall	−0.096	0.009	−10.195	.000

*Note:* All tests are two-tailed, and all standard errors are clustered at the respondent level.

ACME baseline levels for attributes: Agent = Foreign country; Method = Cyberattack; Motivation = Revenge; Outcome = No damage; Target = Military facility.

## Appendix B. Country-Level Results

Appendix B contains the disaggregated country-level AMCE results for analyses appearing in figure 3. Full results associated with the marginal means analyses can be found in Supplementary Material section 3.

**Table B1.** Average marginal component effect for US sample.

Attribute	Level	Estimate	Std. err	z value	*p*
Agent	Hackers with foreign government ties	0.033	0.017	1.908	.056
Agent	Jihadi Islamic group	−0.009	0.017	−0.532	.595
Agent	Organization with foreign government ties	0.007	0.018	0.410	.682
Agent	Unaffiliated group of hackers	−0.022	0.019	−1.179	.239
Agent	Individual hacker	−0.034	0.019	−1.759	.079
Agent	Unknown actor	−0.086	0.019	−4.425	.000
Method	Attack	−0.103	0.016	−6.245	.000
Method	USB	−0.027	0.016	−1.722	.085
Method	Phishing	−0.012	0.015	−0.757	.449
Method	Virus	0.001	0.015	0.036	.972
Motivation	Government overthrow	0.041	0.014	2.864	.004
Motivation	Change government policy	0.010	0.015	0.627	.531
Motivation	Unknown	−0.033	0.015	−2.251	.024
Outcome	Major explosion	0.015	0.023	0.634	.526
Outcome	Theft and release of sensitive information	0.049	0.016	3.119	.002
Outcome	Minor explosion	0.021	0.022	0.957	.338
Outcome	Theft of millions of dollars	0.023	0.016	1.497	.135
Target	Databases	−0.013	0.018	−0.693	.488
Target	Government office building	−0.045	0.016	−2.734	.006
Target	Power station	−0.034	0.017	−2.009	.044
Target	Shopping mall	−0.052	0.016	−3.157	.002

*Note:* All tests are two-tailed, and all standard errors are clustered at the respondent level.

ACME baseline levels for attributes: Agent = Foreign country; Method = Cyberattack; Motivation = Revenge; Outcome = No damage; Target = Military facility.

**Table B2.** Average marginal component effect for UK sample.

Attribute	Level	Estimate	Std. err	z value	*p*
Agent	Hackers with foreign government ties	0.040	0.018	2.260	.024
Agent	Jihadi Islamic group	0.047	0.018	2.580	.010
Agent	Organization with foreign government ties	0.013	0.019	0.707	.480
Agent	Unaffiliated group of hackers	−0.052	0.020	−2.630	.009
Agent	Individual hacker	−0.070	0.020	−3.417	.001
Agent	Unknown actor	−0.098	0.020	−4.935	.000
Method	Attack	−0.175	0.018	−9.785	.000
Method	USB	0.018	0.016	1.099	.272
Method	Phishing	0.036	0.016	2.264	.024
Method	Virus	0.043	0.016	2.726	.006
Motivation	Government overthrow	0.116	0.015	7.859	.000
Motivation	Change government policy	0.097	0.015	6.292	.000
Motivation	Unknown	−0.023	0.016	−1.460	.144
Outcome	Major explosion	0.099	0.022	4.414	.000
Outcome	Theft and release of sensitive information	0.133	0.016	8.369	.000
Outcome	Minor explosion	0.079	0.023	3.386	.001
Outcome	Theft of millions of dollars	0.054	0.016	3.278	.001
Target	Databases	−0.018	0.019	−0.906	.365
Target	Government office building	−0.026	0.015	−1.718	.086
Target	Power station	−0.048	0.015	−3.120	.002
Target	Shopping mall	−0.103	0.016	−6.361	.000

*Note:* All tests are two-tailed, and all standard errors are clustered at the respondent level.

ACME baseline levels for attributes: Agent = Foreign country; Method = Cyberattack; Motivation = Revenge; Outcome = No damage; Target = Military facility.

**Table B3.** Average marginal component effect for Israel sample.

Attribute	Level	Estimate	Std. err	z value	*p*
Agent	Hackers with foreign government ties	0.040	0.018	2.260	.024
Agent	Jihadi Islamic group	0.040	0.018	2.259	.024
Agent	Organization with foreign government ties	0.005	0.018	0.290	.772
Agent	Unaffiliated group of hackers	−0.050	0.019	−2.674	.008
Agent	Individual hacker	−0.117	0.021	−5.597	.000
Agent	Unknown actor	−0.075	0.020	−3.843	.000
Method	Attack	−0.109	0.018	−6.244	.000
Method	USB	−0.030	0.017	−1.779	.075
Method	Phishing	0.014	0.016	0.899	.369
Method	Virus	0.041	0.015	2.714	.007
Motivation	Government overthrow	0.045	0.014	3.197	.001
Motivation	Change government policy	0.023	0.014	1.567	.117
Motivation	Unknown	−0.039	0.015	−2.636	.008
Outcome	Major explosion	0.056	0.022	2.574	.010
Outcome	Theft and release of sensitive information	0.094	0.015	6.380	.000
Outcome	Minor explosion	0.013	0.022	0.591	.555
Outcome	Theft of millions of dollars	0.019	0.016	1.200	.230
Target	Databases	−0.034	0.017	−1.955	.051
Target	Government office building	−0.033	0.015	−2.177	.029
Target	Power station	−0.039	0.015	−2.596	.009
Target	Shopping mall	−0.130	0.016	−7.914	.000

*Note:* All tests are two-tailed, and all standard errors are clustered at the respondent level.

ACME baseline levels for attributes: Agent = Foreign country; Method = Cyberattack; Motivation = Revenge; Outcome = No damage; Target = Military facility.
