# Supplementary material for: Public Opinion and Cyberterrorism
Source: Public Opin Q. 2023 Apr 3;87(1):92–119. doi: 10.1093/poq/nfad006 (PMC10127534; doi:10.1093/poq/nfad006)
Supplement: nfad006_Supplementary_Data [file nfad006_supplementary_data.pdf]

Public Opinion & Cyber-Terrorism

Supplementary Material

Ryan Shandler
University of Oxford
Radcliffe Observatory Quarter, Woodstock Road, Oxford OX2 6GG, England
ryan.shandler@bsg.ox.ac.uk

Nadiya Kostuk
Georgia Institute of Technology
685 Cherry St NW, Atlanta, GA 30313, United States
nkostyuk3@gatech.edu

Harry Oppenheimer
Harvard University
1737 Cambridge St. Cambridge, MA 02138, United States
hoppenheimer@g.harvard.edu

Contents

1 Codebook **2**

2 Data Collection and Sample **4**
2.1 Recruitment Strategy and Sample Quality 4
2.2 Israel 6
2.3 United Kingdom 10
2.4 United States 14

3 Country-Level Results **18**

4 Interaction Effects **22**

5 Conjoint Assumptions **27**

6 References **31**

1 Codebook

Original variables in dataset ("Combined Conjoint Data.csv")

Table S1: Survey Codebook

Variable Name	Data Points
CountryCode	1 = US; 2 = UK; 3 = Israel
RandomNumber	Ranging from 10000 to 999999
Scenario1... thru to Scenario7	1 = cyberterrorism; 2 = NOT cyberterrorism;
Method1... thru to Method7	1. [blank] (*) 2. using a computer virus 3. Phishing 4. Infected USB 5. Cyber attack
Target1... thru to Target7	1. an electricity power station 2. a military facility (*) 3. a shopping mall 4. a government office building 5. online databases (containing personal information about citizens)
Agent1... thru to Agent7	1. No casualties or property damage were reported in the attack (*) 2. Minor Explosion 3. Major Explosion 4. Millions of dollars were stolen in the attack 5. Sensitive personal and organizational data was stolen and publicly released in the attack
Motivation1... thru to Motivation7	1. a foreign country (*) 2. an organization with ties to a foreign government 3. a group of hackers with ties to a foreign government 4. an unaffiliated group of hackers 5. an individual hacker 6. an unknown actor 7. a Jihadi Islamic group.
Outcome1... thru to Outcome7	1. ... the motivation for the incident remains unknown. 2. ... the incident was motivated by the goal of overthrowing the government. 3. ... the incident was motivated by the goal of changing government policy. 4. ... the incident was motivated by revenge. (*)
Comp_Literacy1	4 = correct answer
Comp_Literacy2	1 = correct answer
Comp_Literacy3	4 = correct answer
Comp_courses	1 = "I've never taken any of these courses."; 2 = "I've taken 1 course."; 3 = "I've taken between 2 - 5 courses."; 4 = "I've taken more than 5 courses."
Political_position	1 = Extremely left; 6 = Extremely Right
Party_ID_US	1 = Strong Democrat; 7 = Strong Republican
Prev_Exposure_Terror_1	0 = no; 1 = yes
Prev_Exposure_Terror_2	0 = no; 1 = yes
Prev_Exposure_Cyber_1	0 = no; 1 = yes
Prev_Exposure_Cyber_2	0 = no; 1 = yes
Prev_Exposure_Cyber_3	0 = no; 1 = yes
Personal_Threat_1	1 = not at all; 6 = extremely
Personal_Threat_2	1 = not at all; 6 = extremely
Personal_Threat_3	1 = not at all; 6 = extremely
National_Threat_1	1 = not at all; 6 = extremely
National_Threat_2	1 = not at all; 6 = extremely
National_Threat_3	1 = not at all; 6 = extremely

Table S2: Survey Codebook (continued)

Variable Name	Data Points
Gender	1 = female; 2 = male, 3 = other
Marital_Status	1. Married or civil union; 2. Single, never married; 3 Divorced; 4. Separated; 5. Widowed; 6. In a relationship; 7. Decline to answer
Children	1. None; 2 One; 3. Two; 4. Three; 5. Four or more; 6. Decline to answer
Education	1. Less than high school; 2. Completed some high school; 3. High school graduate or equivalent (e.g. GED); 4. Jpb-specific training programs after high school; 5. Completed some college, but no degree; 6. Associate's Degree; 7. College graduate (e.g. B.A., A.B., B.S.); 8. Completed some graduate school, but no degree; 9. Completed graduate school (e.g. M.S., M.D., Ph.D.)
Employment	1. Employed full time; 2. Employed part time; 3. Self-employed; 4. Temporarily laid off due to COVID-19; 5. Not employed, but looking for work; 6. Not employed and not looking for work; 7. Not employed, unable to work due to a disability or illness; 8. Retired; 9. Student; 10. Homemaker; 11. Decline to answer
Religion	1. Protestan; 2. Catholic; 3. Other Christian; 4. Jewish; 5. Muslim; 6. Hindu; 7. Buddhist; 8. Other faith; 9. Unaffiliated; 10. Decline to answer
Income	1. Much lower than average; 2. Lower than average; 3. A little lower than average; 4. Average; 5. A little higher than average; 6. Higher than average; 7. Much higher than average; 8. Decline to answer
COVID19_1	0 = no; 1 = yes
COVID19_2	0 = no; 1 = yes
COVID19_3	0 = no; 1 = yes
COVID19_4	0 = no; 1 = yes
Consent	1 = Yes; 2 = No

2 Data Collection and Sample

2.1 Recruitment Strategy and Sample Quality

The primary objective of our study is to estimate the causal effects of specific incident attributes on the ascription of an attack as cyber-terrorism. Given this, we employ a design where respondents are assigned to appraise scenarios with randomly generated attributes. We draw from a large sample of respondents, across three countries, who are accessed through online survey companies that employ a non-probability sampling method. To account for any demographic imbalances that would undermine any inferences of causality, we conduct balance checks that test whether specific demographic and attitudinal covariates (gender and political ideology) predict the assignment of the study's independent variable (attack attributes). These analyses appear in Figures S2, S3, S5, S6, S8, and S9, and confirm that the random assignment was successful.

To reach the participants, we employed three different survey panels due to our preference for providers that have large market share and a diverse sample of participants in each country. In the United States, we drew our sample from Amazon Mechanical Turk (MTurk), in the United Kingdom we used the Prolific, and in Israel we worked with Midgam. All three providers are frequently used in social science research. While unmoderated survey panels such as MTurk and Prolific have a mixed reputation regarding the representativeness of their participant pools, research has shown that these two panels consistently outperformed laboratory-based convenience samples in ensuring variation across socio-demographic and political characteristics of interest (Berinsky et al., 2012; Hauser and Schwarz, 2016; Palan and Schitter, 2018). The Israeli survey provider − Midgam − is moderated by a team of facilitators, with access to more than 90,000 Israeli respondents (more than 1% of the entire Israeli population).

Respondents accessed via the un-moderated Internet survey companies (MTurk and Prolific) were required to have an approval rating of at least 96%, have completed at least 50 surveys, be a resident of the particular country, and be over 18 years of age. These quality checks are not required with Midgam, where the studies are distributed by dedicated technicians to verified users. In the Israeli sample, we limited the sample to Jewish respondents, since attacks against national institutions are unlikely to be viewed as an attack against the in-group by some portion of Palestinian respondents.

A reCaptcha test was inserted at the beginning of all surveys to weed out bots. The study was

fielded simultaneously on 26 - 27 August, 2020, during which time we recruited 3,242 respondents (United States, N = 1,077; United Kingdom, N = 1,042; Israel, N = 1,123. We retain only the 3,036 respondents (United States, N = 1,012; United Kingdom, N = 1,010; Israel, N = 1,014) who completed all of the conjoint questions and who passed the reCapctha test. This amounts to a total non-completion rate of 6.35% — a rate that compares favorably to completion metrics in other web-based surveys (Liu and Wronski, 2018). As is the norm with online survey panels, our respondents tended to be young, with a median age of 38. Following best practice, we refrain from removing "speeders" who complete the survey particularly fast, since their removal does not alter marginal distributions in a meaningful way, and such paticipants are representative of a segment of the larger population who only partially absorb data before reaching political decision (Greszki et al., 2015).

In Sections 2.2-2.4, we review the demographic breakdown of each country's sample, and we conduct balance checks to confirm that no demographic or attitudinal covariates predict the assignment of the study's independent variable.

2.2 Israel

The sample for Israel was collected using Midgam. 1,014 individuals completed the survey on August 26, 2020.

Table S3: Israel survey sample characteristics (n=1,014)

Gender	
Male	0.507
Female	0.493
Age	
18-29	0.257
30-44	0.383
45-64	0.271
65+	0.089
Education	
Elementary school	0.018
Middle school	0.007
High school graduate	0.220
Job-specific training programs after high school	0.089
Partial completion of BA	0.136
BA	0.348
MA	0.389
PhD	0.022
Political Position	
Extremely left	0.009
Liberal	0.064
Slightly liberal	0.197
Slightly conservative	0.389
Conservative	0.288
Extremely Right	0.053

Figure S1: Israel survey sample treatment balance

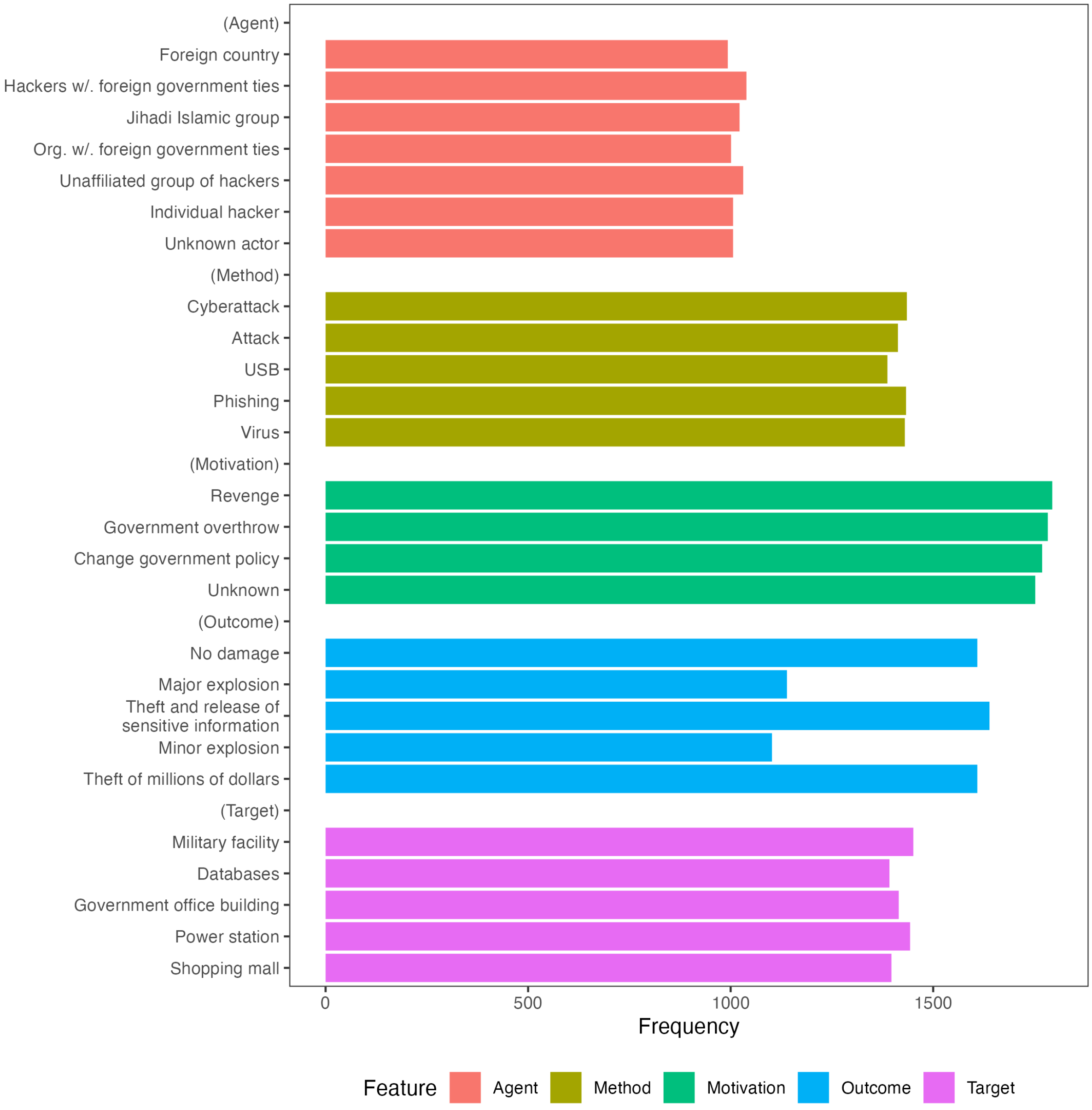

Figure S2: Israel survey sample gender balance

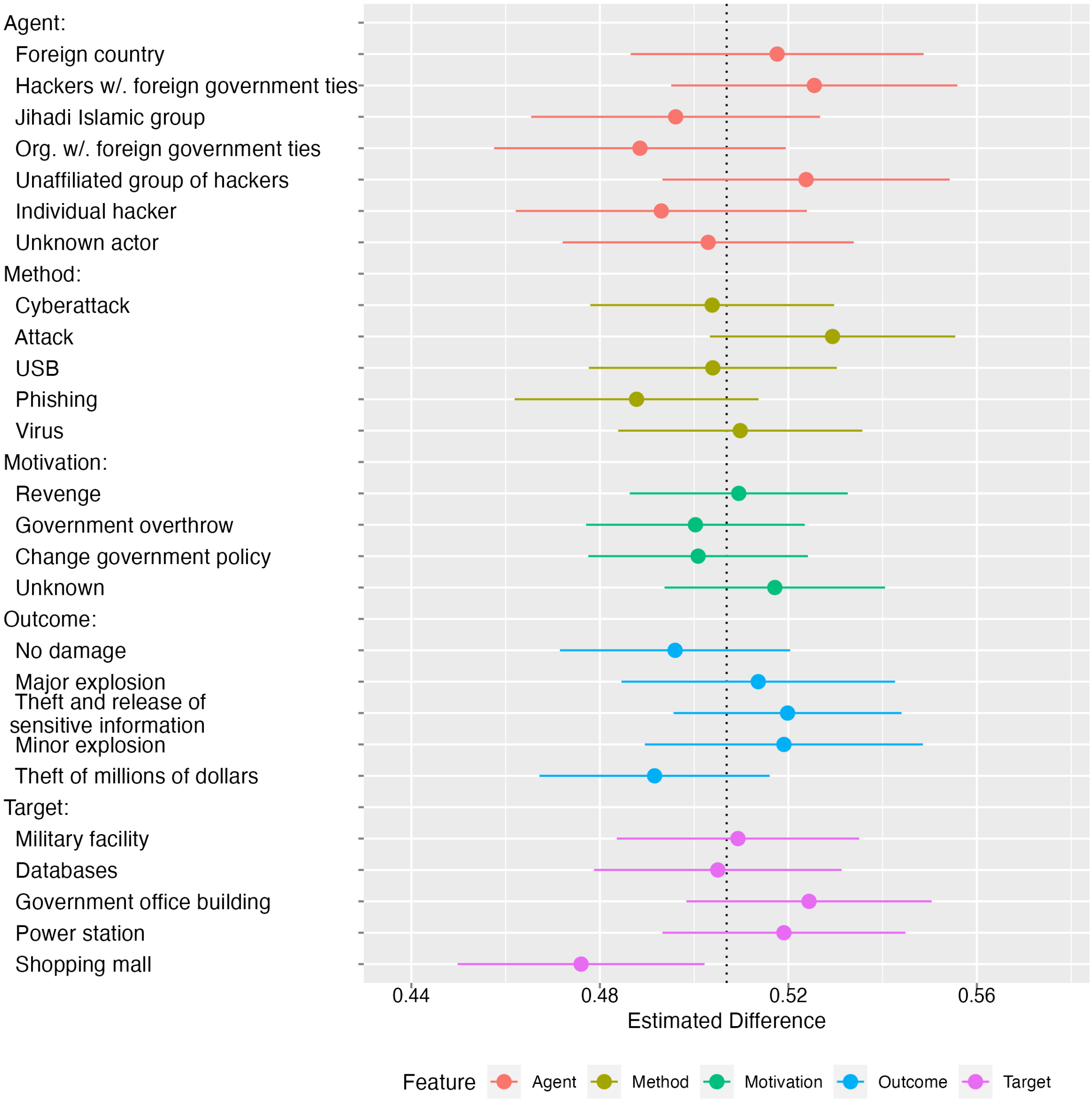

Figure S3: Israel survey sample political ideology balance

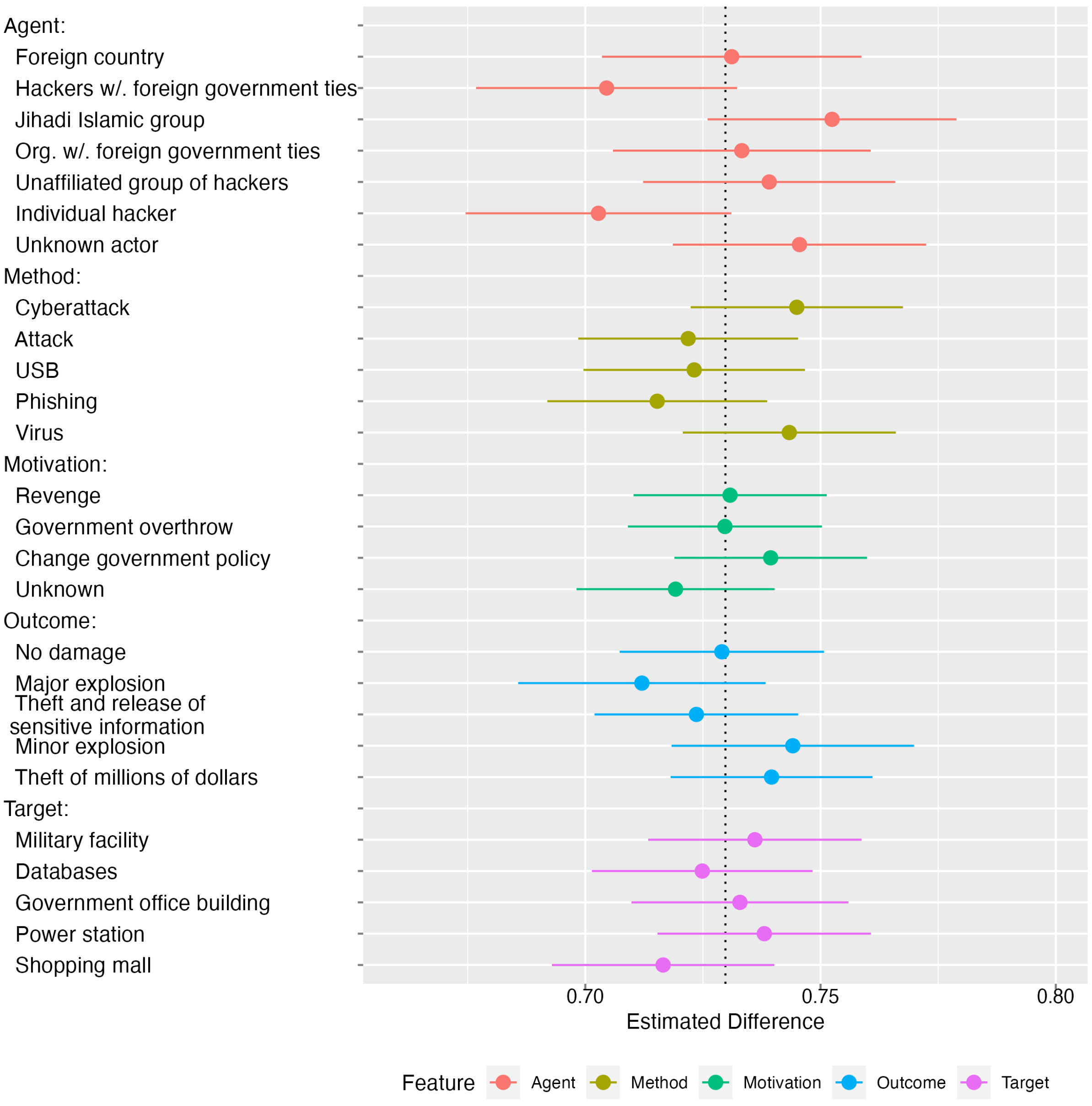

This figure contains a plot of conservative ideology by treatment category with 95% confidence intervals. Conservative indicates that the respondents answered either a 5 or a 6 on our ideology measure, indicating that they were either “conservative” or “very conservative/right”.

2.3 United Kingdom

The sample for the United Kingdom was collected using Prolific. 1,010 individuals completed the survey on August 26, 2020.

Table S4: UK survey sample characteristics (n=1,010)

Gender	
Male	0.499
Female	0.501
**Age**	
18-29	0.417
30-44	0.340
45-64	0.213
65+	0.031
**Education**	
Less than secondary	0.002
Completed some secondary	0.012
Completed secondary	0.048
Post-secondary non-university education	0.157
Short-cycle tertiary education (1 year)	0.025
Partial completion of university	0.097
University graduate	0.389
Completed some graduate school, but no degree	0.009
Completed a graduate degree (e.g. M.S., M.D., Ph.D.)	0.161
**Political Position**	
Extremely left	0.058
Liberal	0.200
Slightly liberal	0.410
Slightly conservative	0.287
Conservative	0.036
Extremely Right	0.009

Figure S4: UK survey sample treatment balance

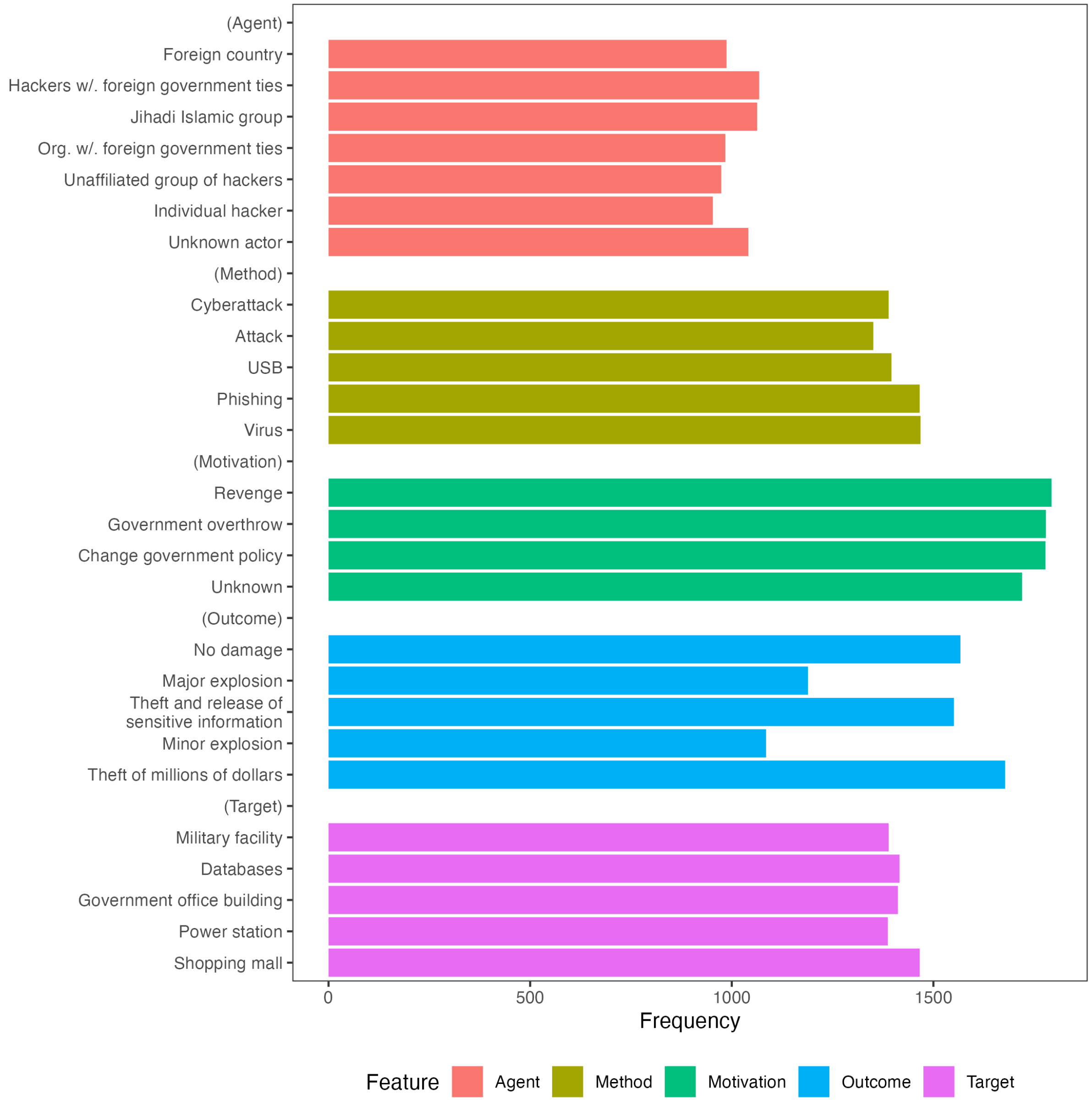

Figure S5: UK survey sample gender balance

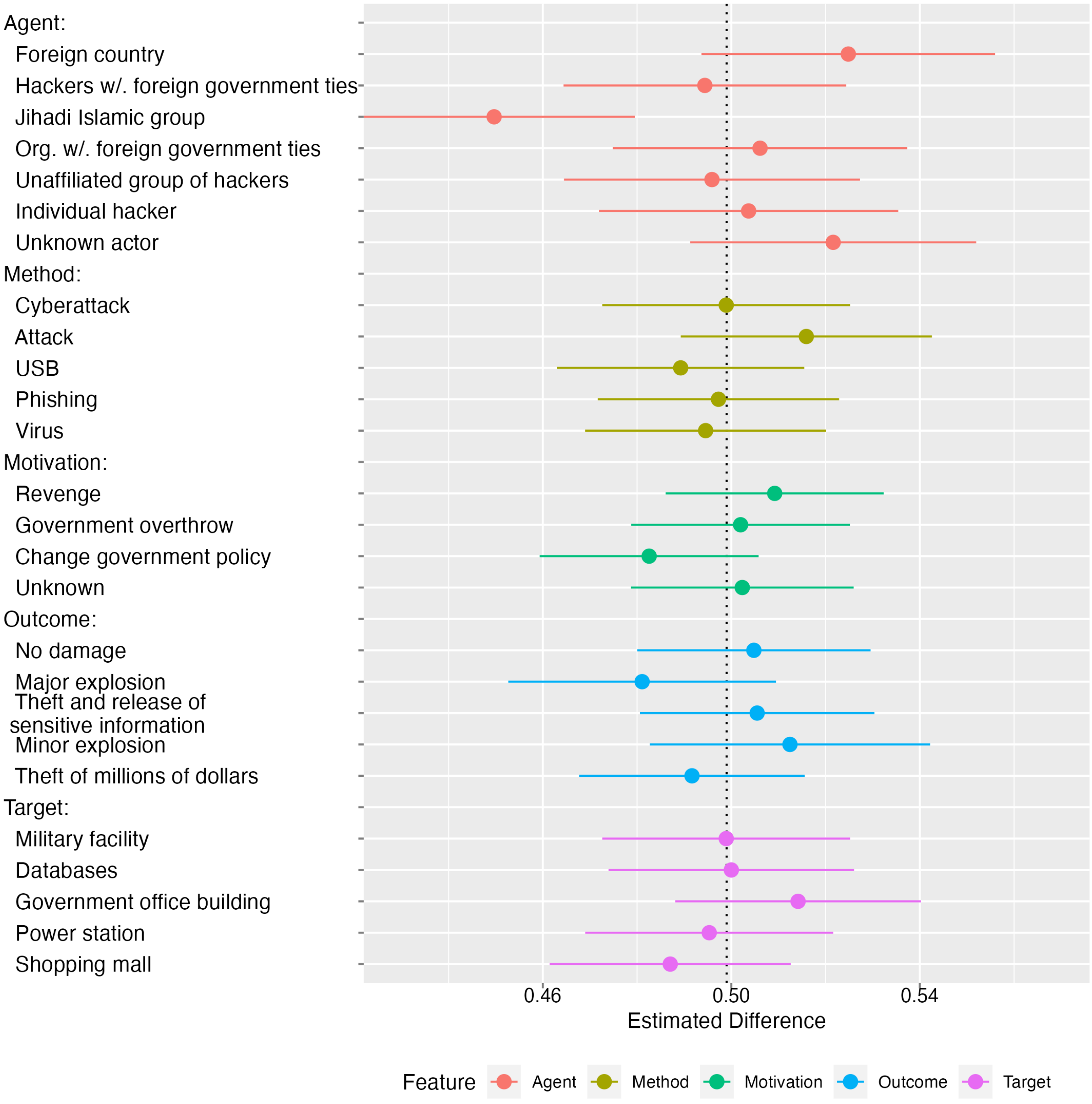

Figure S6: UK survey sample political ideology balance

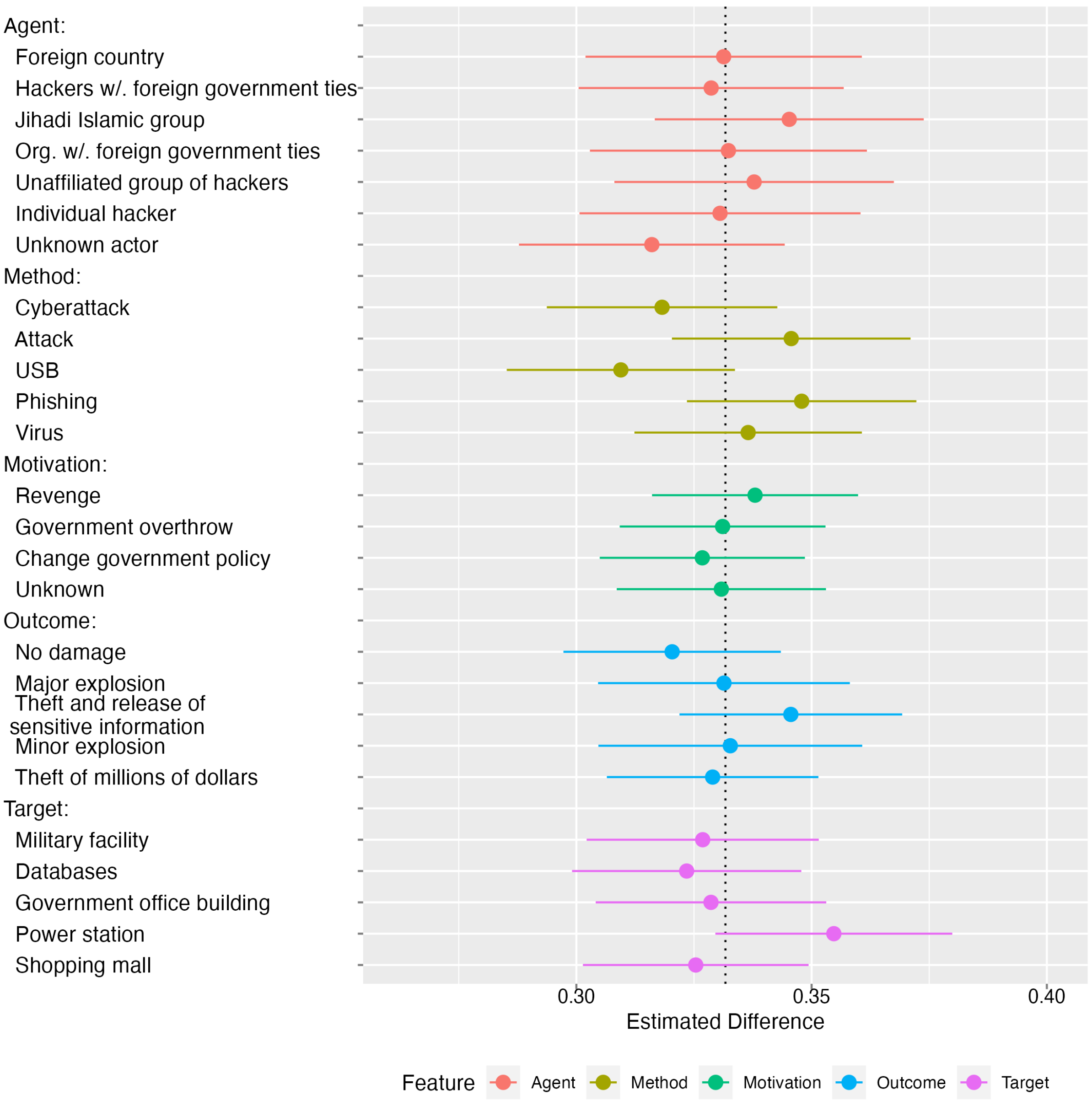

This figure contains a plot of conservative ideology by treatment category with 95% confidence intervals. Conservative indicates that the respondents answered either a 5 or a 6 on our ideology measure, indicating that they were either "conservative" or "very conservative/right".

2.4 United States

The sample for the United States was collected using Mechanical Turk (MTurk). 1,012 individuals completed the survey between August 25 and August 26, 2020.

Table S5: US survey sample characteristics (n=1,012)

Gender	
Male	0.638
Female	0.362
Age	
18-29	0.219
30-44	0.545
45-64	0.210
65+	0.025
Education	
Less than high school	0.002
Completed some high school	0.007
High school graduate or equivalent (e.g. GED)	0.053
Job-specific training programs after high school	0.010
Completed some college, but no degree	0.088
Associate's Degree	0.010
College graduate (e.g. B.A., A.B., B.S.)	0.524
Completed some graduate school, but no degree	0.027
Completed graduate school (e.g. M.S., M.D., Ph.D.)	0.190
Party Identification	
Strong Democrat	0.189
Weak Democrat	0.067
Independent Democrat	0.076
Independent	0.144
Independent Republican	0.118
Weak Republican	0.116
Strong Republican	0.291

Figure S7: US survey sample treatment balance

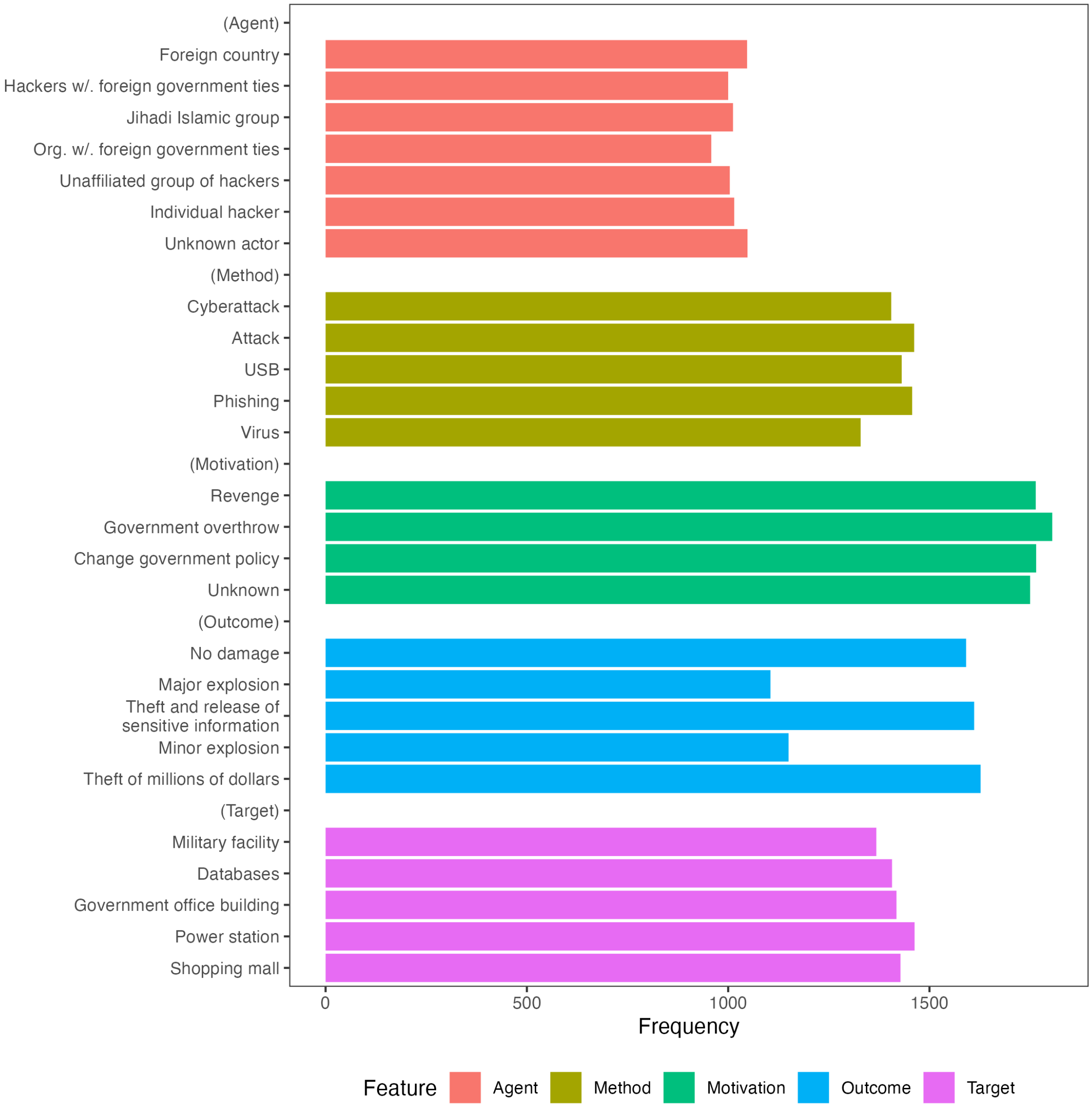

Figure S8: US survey sample gender balance

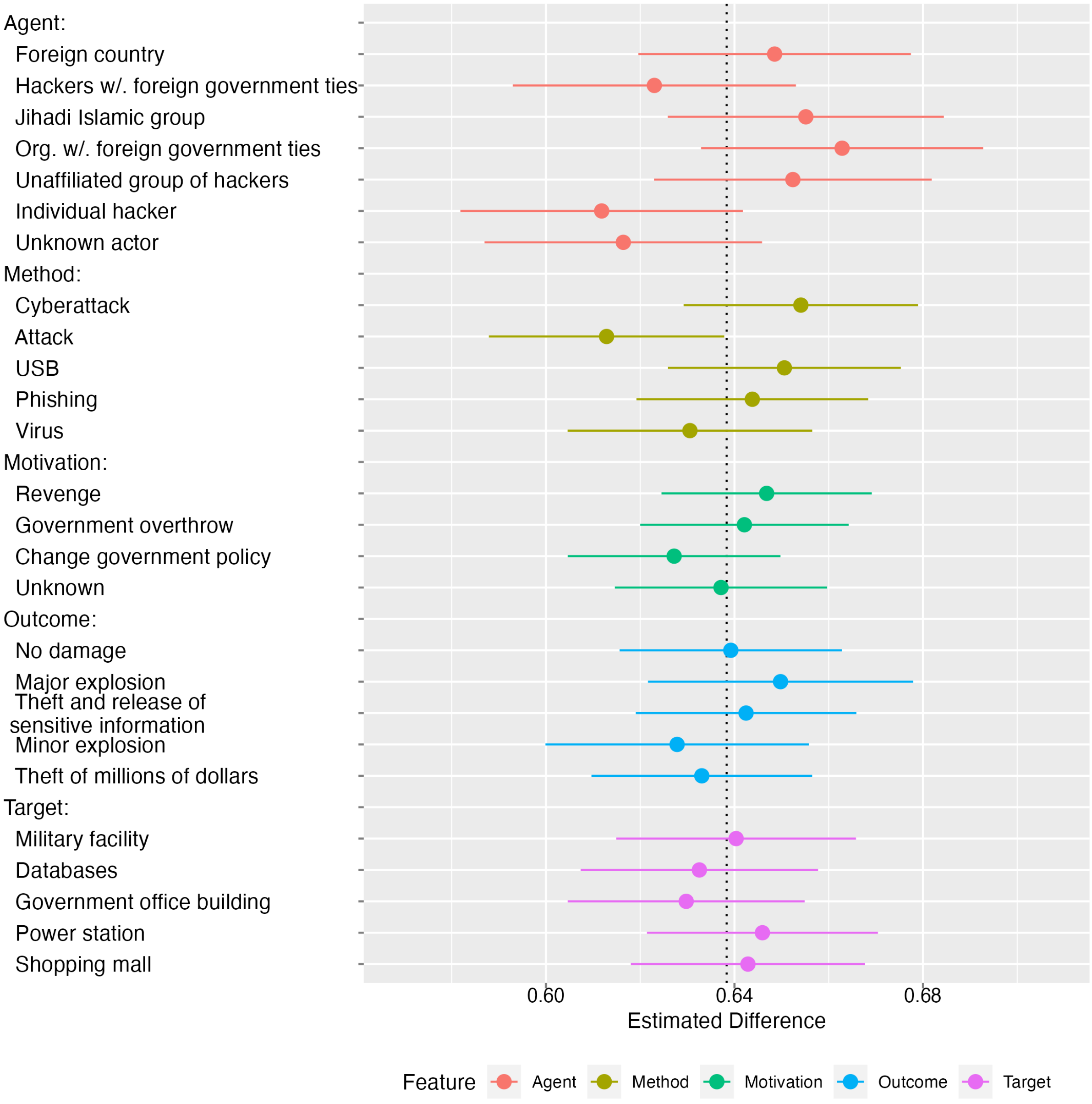

Figure S9: US survey sample political ideology balance

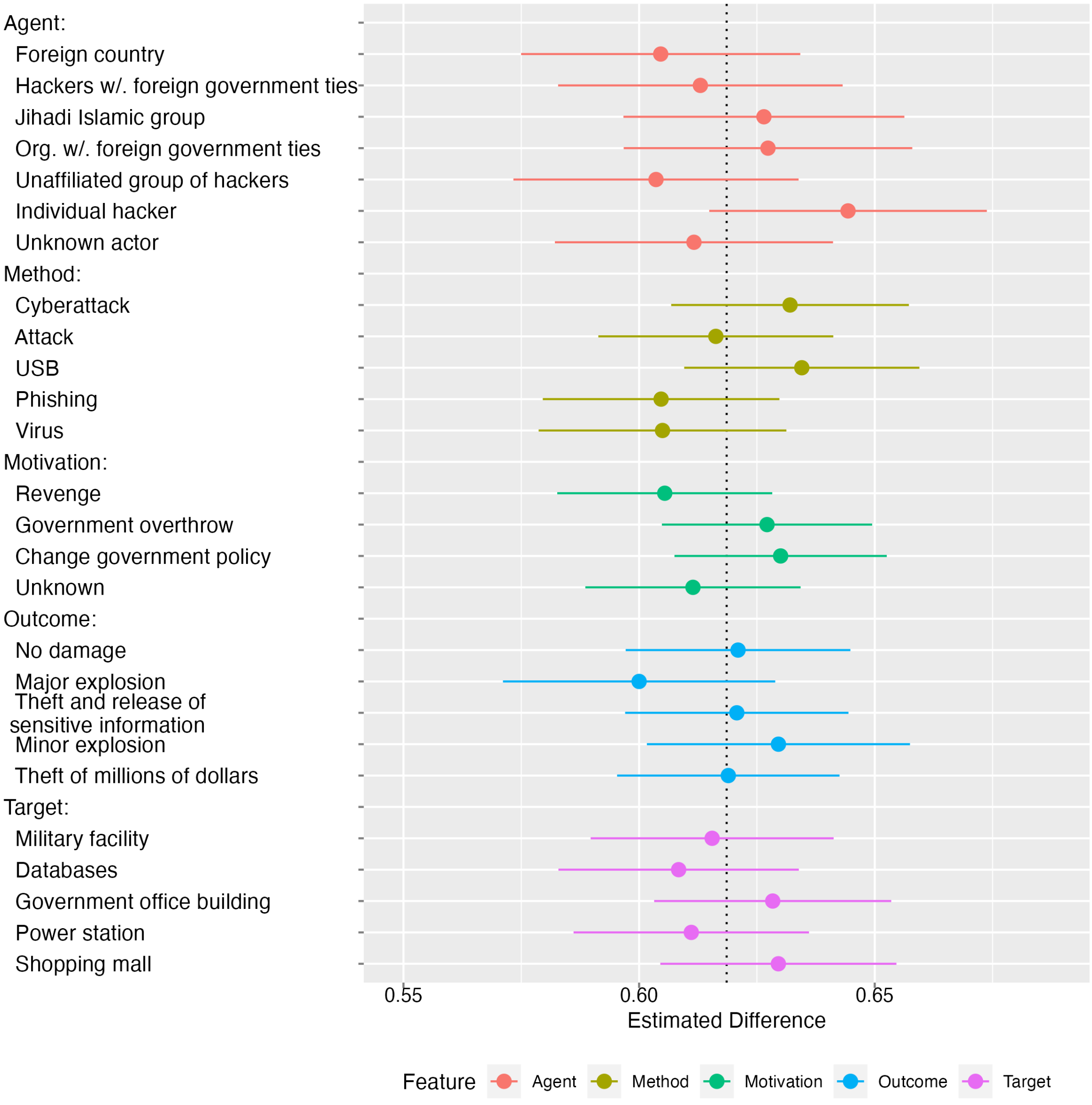

This figure contains a plot of conservative ideology by treatment category with 95% confidence intervals. Conservative indicates that the respondents answered either a 5 or a 6 on our ideology measure, indicating that they were either "conservative" or "very conservative/right".

3 Country-Level Results

In the main manuscript and accompanying appendices, we disaggregate our conjoint analysis by country, and compare the country-level differences by using the AMCE value as the means of comparison. Here, we supplement this analysis by using marginal means. The significant marginal means differences across countries are plotted in Figure S10, and fully specified in Tables S6-S8.

Figure S10: Marginal Means Difference by Country

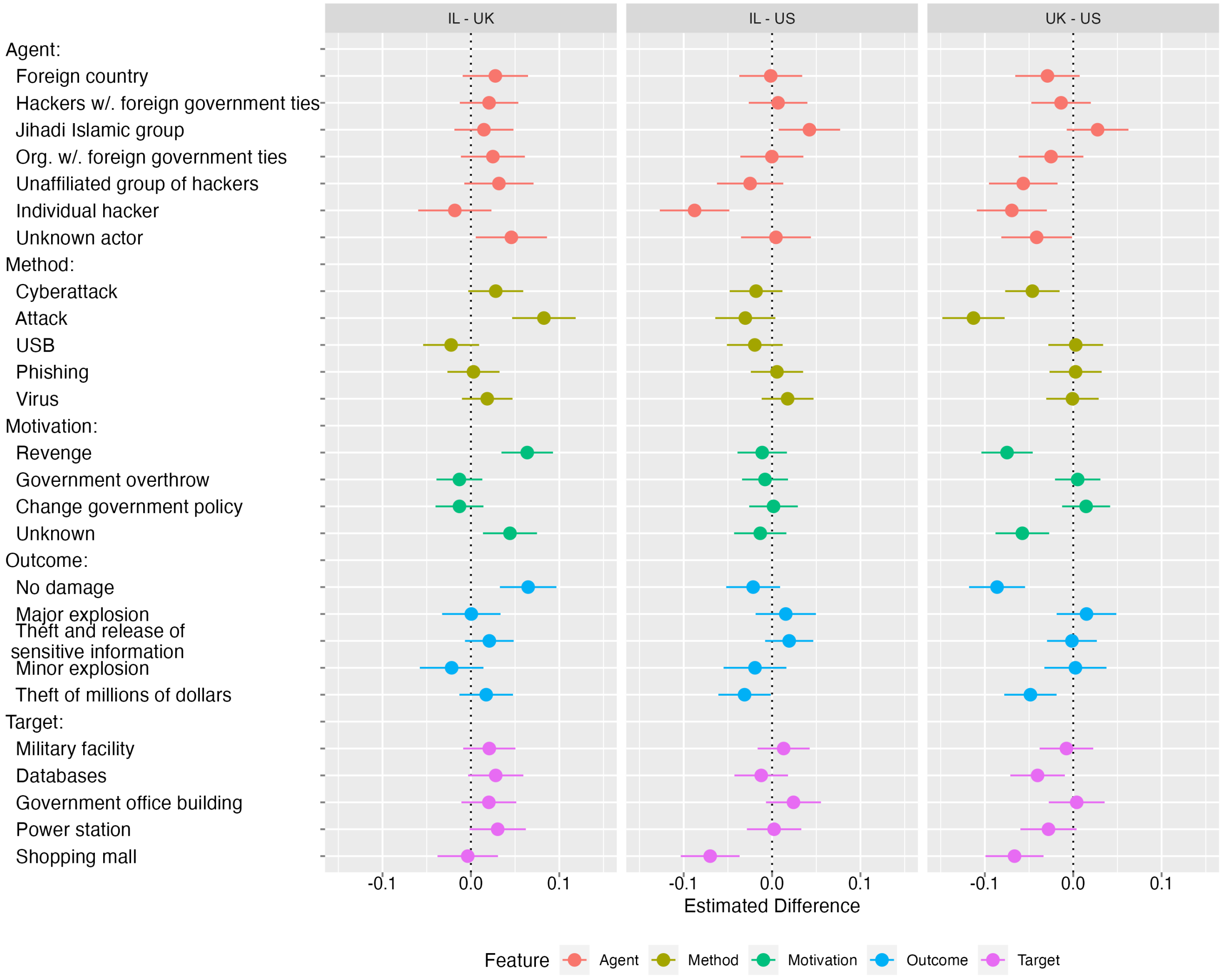

This figure contains a plot of the difference in marginal mean levels cyberterrorism attribution by treatment category with 95% confidence intervals. This presents the difference by country for all respondents. For example, attributing the motivation to revenge has a more positive effect on attribution for American respondents than Israeli or British respondents regardless of ideology.

Table S6: Marginal Means Difference (IL-UK)

Attribute	Level	Estimate	Std. Err	z value	Pr(> $\|z\|$)	
Agent	Unknown actor	0.0458	0.0206	2.2270	0.0259	*
Agent	Individual hacker	-0.0182	0.0212	-0.8610	0.3893	
Agent	Unaffiliated group of hackers	0.0317	0.0200	1.5856	0.1128	
Agent	Org. w/. foreign government ties	0.0249	0.0185	1.3456	0.1784	
Agent	Jihadi Islamic group	0.0147	0.0171	0.8637	0.3877	
Agent	Hackers w/. foreign government ties	0.0206	0.0169	1.2166	0.2238	
Agent	Foreign country	0.0276	0.0189	1.4660	0.1427	
Method	Virus	0.0185	0.0146	1.2640	0.2062	
Method	Phishing	0.0029	0.0150	0.1918	0.8479	
Method	USB	-0.0224	0.0162	-1.3849	0.1661	
Method	Attack	0.0826	0.0184	4.50150	0.0000	***
Method	Cyberattack	0.0281	0.0159	1.7689	0.0769	
Motivation	Unknown	0.0442	0.0156	2.8332	0.0046	**
Motivation	Change government policy	-0.0129	0.0138	-0.9352	0.3497	
Motivation	Government overthrow	-0.0130	0.0132	-0.9848	0.3247	
Motivation	Revenge	0.0637	0.0149	4.2843	0.0000	***
Outcome	Theft of millions of dollars	0.0173	0.0155	1.1185	0.2634	
Outcome	Minor explosion	-0.0218	0.0184	-1.1877	0.2349	
Outcome	Theft and release of sensitive information	0.0208	0.0141	1.4818	0.1384	
Outcome	Major explosion	0.0005	0.0169	0.0275	0.9780	
Outcome	No damage	0.0648	0.0163	3.9683	0.0000	***
Target	Shopping mall	-0.0036	0.0175	-0.2061	0.8367	
Target	Power station	0.0303	0.0162	1.8667	0.0619	
Target	Government office building	0.0203	0.0158	1.2813	0.2001	
Target	Databases	0.0281	0.0160	1.7598	0.0785	
Target	Military facility	0.0208	0.0151	1.3793	0.1678	

*Signif. Codes: ***: 0.001, **: 0.01, *: 0.05*
All tests are two-tailed, all standard errors are clustered at the respondent level.

Table S7: Marginal Means Difference (IL-US)

Attribute	Level	Estimate	Std. Err	z value	Pr(> $\|z\|$)	
Agent	Unknown actor	0.0044	0.0202	0.2176	0.8278	
Agent	Individual hacker	-0.0877	0.0201	-4.3664	0.0000	***
Agent	Unaffiliated group of hackers	-0.0249	0.0192	-1.2980	0.1943	
Agent	Org. w/. foreign government ties	-0.0002	0.0182	-0.0136	0.9892	
Agent	Jihadi Islamic group	0.0423	0.0177	2.3855	0.0171	*
Agent	Hackers w/. foreign government ties	0.0068	0.0169	0.4012	0.6883	
Agent	Foreign country	-0.0015	0.0182	-0.0833	0.9336	
Method	Virus	0.0176	0.0150	1.1741	0.2403	
Method	Phishing	0.0055	0.0151	0.3671	0.7135	
Method	USB	-0.0196	0.0161	-1.2155	0.2242	
Method	Attack	-0.0303	0.0174	-1.7449	0.0810	
Method	Cyberattack	-0.0181	0.0152	-1.1901	0.2340	
Motivation	Unknown	-0.0134	0.0151	-0.8872	0.3750	
Motivation	Change government policy	0.0016	0.0140	0.1168	0.9070	
Motivation	Government overthrow	-0.0080	0.0133	-0.6018	0.5473	
Motivation	Revenge	-0.0112	0.0143	-0.7832	-0.7832	
Outcome	Theft of millions of dollars	-0.0312	0.0151	-2.0617	0.0392	*
Outcome	Minor explosion	-0.0193	0.0181	-1.0661	0.2864	
Outcome	Theft and release of sensitive information	0.0194	0.0139	1.3927	0.1637	
Outcome	Major explosion	0.0155	0.0174	0.8897	0.3736	
Outcome	No damage	-0.0214	0.0156	-1.3769	0.1686	
Target	Shopping mall	-0.0700	0.0170	-4.1210	0.0000	***
Target	Power station	0.0023	0.0157	0.1455	0.8843	
Target	Government office building	0.0242	0.0159	1.5259	0.1270	
Target	Databases	-0.0123	0.0155	-0.7938	0.4273	
Target	Military facility	0.0131	0.0150	0.8709	0.3838	

*Signif. Codes: ***: 0.001, **: 0.01, *: 0.05*
All tests are two-tailed, all standard errors are clustered at the respondent level.

Table S8: Marginal Means Difference (UK-US)

Attribute	Level	Estimate	Std. Err	z value	Pr(> $\|z\|$)	
Agent	Unknown actor	-0.0414	0.0204	-2.0294	0.0424	
Agent	Individual hacker	-0.0695	0.0202	-3.4362	0.0006	***
Agent	Unaffiliated group of hackers	-0.0566	0.0198	-2.8556	0.0043	**
Agent	Org. w/. foreign government ties	-0.0251	0.0187	-1.3450	0.1786	
Agent	Jihadi Islamic group	0.0276	0.0178	1.5467	0.1219	
Agent	Hackers w/. foreign government ties	-0.0138	0.0172	-0.8008	0.4233	
Agent	Foreign country	-0.0292	0.0186	-1.5674	0.1170	
Method	Virus	-0.0009	0.0151	-0.0578	0.9539	
Method	Phishing	0.0027	0.0151	0.1766	0.8598	
Method	USB	0.0028	0.0158	0.1780	0.8587	
Method	Attack	-0.1129	0.0180	-6.2726	0.0000	***
Method	Cyberattack	-0.0462	0.0157	-2.9406	0.0033	**
Motivation	Unknown	-0.0576	0.0155	-3.7170	0.0002	***
Motivation	Change government policy	0.0146	0.0139	1.0517	0.2930	
Motivation	Government overthrow	0.0051	0.0131	0.3863	0.6993	
Motivation	Revenge	-0.0749	0.0148	-5.0537	0.0000	***
Outcome	Theft of millions of dollars	-0.0485	0.0151	-3.2078	0.0013	**
Outcome	Minor explosion	0.0025	0.0179	0.1379	0.8903	
Outcome	Theft and release of sensitive information	-0.0015	0.0144	-0.1027	0.9182	
Outcome	Major explosion	0.0150	0.0172	0.8715	0.3835	
Outcome	No damage	-0.0862	0.0162	-5.3309	0.0000	***
Target	Shopping mall	-0.0664	0.0168	-3.9663	0.0001	***
Target	Power station	-0.0280	0.0162	-1.7288	0.0838	
Target	Government office building	0.0039	0.0161	0.2430	0.8080	
Target	Databases	-0.0404	0.0158	-2.5622	0.0104	*
Target	Military facility	-0.0077	0.0155	-0.4979	0.6186	

*Signif. Codes: ***: 0.001, **: 0.01, *: 0.05*
All tests are two-tailed, all standard errors are clustered at the respondent level.

4 Interaction Effects

The baseline results described above allowed us to quantify the importance of various attributes to the classification of incidents as cyber-terrorism. Yet our empirical design grants us the ability to conduct more nuanced analyses that measure the interaction between people's personal beliefs and experiences and the ascription of cyber-terrorism. For example, it is well established that people with a right-wing political orientation view security incidents more severely than left-wing oriented people. This manifested in the conjoint survey with right-wing respondents classifying a small yet statistically significantly higher number of incidents as cyber-terrorism than left-wing respondents (77% compared to 74%, $p < .001$).

To test for exogenous interaction effects, we fit a fully interacted model to the pooled data and then conducting an F-test to compare the two models. This process in effect reveals whether the designation of an incident as cyber-terrorism varies across levels of the intervening variable. Following Leeper et al. (2020), we acknowledge that the interaction effects in conjoint analyses are sensitive to reference category choices, and we stand by the justifications for the selection of each reference category described in the main manuscript. In the sections below, we look at two relevant intervening exogenous variables that are likely to influence the perception of a cyber incident as cyber-terrorism.

In Figure S11, we report the effects of incident attributes on the designation of an attack as cyber-terrorism, disaggregated by political orientation. The left-hand panel depicts the estimates of each attribute effect for respondents with self-described conservative political beliefs, and the right-hand panel reflects those participants with liberal political attitudes.[1] What is immediately observable in Figure S11 is that the attack attributes trigger greater volatility among liberal respondents who are more influenced by the attack characteristics—both positively and negatively—than their more steadfast conservative counterparts.[2] We can infer from this that conservative individuals are more comfortable associating cyber-attacks in general with cyber-terrorism, while this association is weaker for liberals and more reliant on the presence or absence of particular features.

A key point of difference in the qualities that spur the designation of an attack as cyber-terrorism between liberals and conservatives is the effect of the announced motivation of the attacker. An-

[1] Political orientation was measured on a self-described six-point scale from extremely liberal to extremely conservative. In accordance with local lingo, the British and Israeli scales referred to left-wing and right-wing rather than liberal and conservative.

[2] This volatility is in reference to the baseline reference categories for each attribute.

nouncing that the attacker's motivations were either 'changing government policy' or 'overthrowing the government' heightened the likelihood that liberal respondents would designate an attack as cyber-terrorism to a significantly greater degree than for conservative respondents (at $p < .00$ and $p < .03$ respectively). This accords with past research showing how people with left-wing political attitudes are more likely to take account and be influenced by terrorists' motivations than people with right-wing orientations (Sánchez and García, 2016). This outcome challenges findings from Canetti et al. (2021) who question the assumption that motive attribution influences public support for counter-terror policy, and raises the possibility that motive attribution might behave differently in cyberspace than in conventional arenas.

Another key point of difference emerges in response to the outcome of the attack. The marginal component effects of the 'major explosion' outcome and the 'theft and dissemination of sensitive information' outcome were significantly different for liberal and conservative respondents, at $p < .02$ for both effects, with liberal respondents associating these outcomes with cyber-terrorism at significantly higher levels than conservative respondents, who were less swayed by such features.

Figure S11: *Effects of Incident Attributes on Probability of Being Classified as Cyber-Terrorism—By Political Orientation*

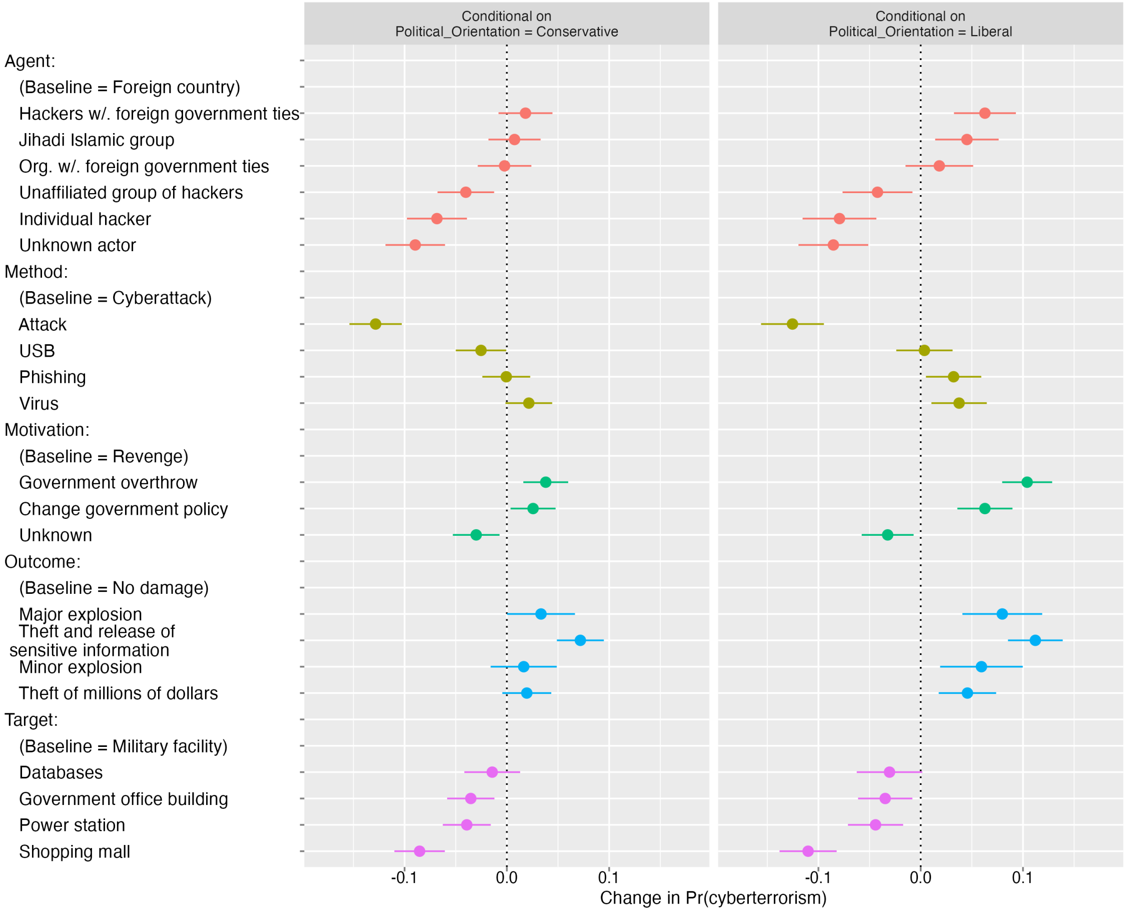

Note: This plot depicts estimates of the effects of each randomly assigned attribute values on the probability of a scenario being classified as cyber-terrorism. Estimates are based on the AMCE model with clustered standard errors. Bars represent 95% confidence intervals. The uppermost values in each attribute, which lack confidence intervals, reflects the reference category for each attribute against which the estimates are based. This plot reveals a pooled estimate for liberal and conservative respondents drawn from the full multi-country sample.

Past research has persuasively identified the role of cyber-domain expertise in understanding the response of the general public to cyber attacks in general (Kostyuk and Wayne, 2020). As such, it behooves us to examine the intervening effect of computer literacy upon the classification of cyber acts as cyber-terrorism.[3] Figure S12 visualizes the distribution of attribute effects between respondents designated as high computer literacy (left-hand panel) and low computer literacy (right-

[3]Cyber literacy was measured using a three-item summative scale adapted from Kostyuk and Wayne (2020) that asked respondents to identify the meaning of digital terminology such as trojan horses, data packets, and spam email.

hand panel).

Several sets of attributes are found to be statistically significantly different in the AMCEs of this sub-group. First, the AMCEs of three of the outcome attribute values (major explosion, minor explosion, theft and dissemination of sensitive information) are significantly different for high and low computer literacy, at $p < .000$, $p < .000$ and $p < .048$ respectively, with high computer literacy respondents associating incidents with each of these outcomes with cyber-terrorism at significantly high levels than low-computer literacy respondents. This logically follows the idea that members of the public with domain expertise will better perceive the rarity and gravity of a cyber-driven explosion, and also the potential significance of data as a valuable target for adversaries.

In a similar vein, we can also see that the AMCE of 'individual hacker' is significantly different for high and low computer literacy respondents, at $p < .001$, with high computer literacy respondents associating attacks from individual actors with cyber-terrorism at significantly lower levels than low-computer literacy respondents. Again, this makes sense given that those with some level of digital proficiency will understand the potential limitations that hinder the ability of individual hackers from causing substantial damage (Kostyuk and Wayne, 2020).

It is interesting to note that there was no difference between the relative marginal component effect of the two groups with respect to cyber-attack methodologies. High computer literacy respondents showed no inclination to view any particular method of attack with greater wariness than their low-computer literacy counterparts.

Figure S12: *Effects of Incident Attributes on Probability of Being Classified as Cyber-Terrorism—By Computer Literacy*

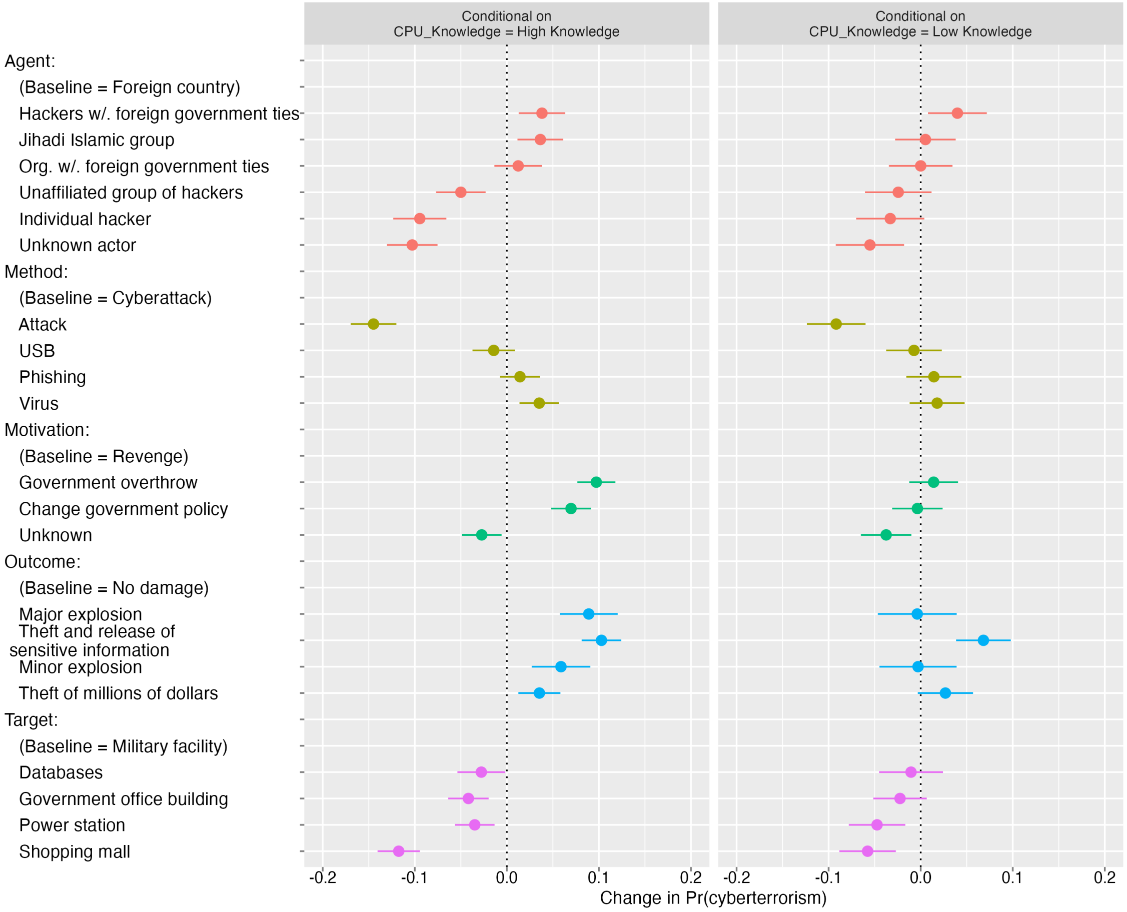

Note: This plot depicts estimates of the effects of each randomly assigned attribute values on the probability of a scenario being classified as cyber-terrorism. Estimates are based on the AMCE model with clustered standard errors. Bars represent 95% confidence intervals. The uppermost values in each attribute, which lack confidence intervals, reflects the reference category for each attribute against which the estimates are based. This plot reveals a pooled estimate for respondents with high and low computer literacy drawn from the full multi-country sample.

5 Conjoint Assumptions

As Hainmueller et al. (2014) note, conjoint experiments assume both *no profile order effects* and *stability and no carryover*. Our experimental design presents respondents with individual scenarios and asks them to decide whether it contains an act of cyber-terrorism, similar to other ratings-based conjoint designs. Randomizing the order of the scenario text is not feasible in our study since we present a vignette (instead of tabular set) to respondents. Randomizing the order of sentences in a paragraph vignette would interfere with its logical coherence. However, stability and no carryover remains an important assumption to test. Table S9 presents the cyber-terrorism variable as a function of the response round (baseline 3rd round) with fixed effects for the respondent. Respondents were more likely to assign the cyberterrorism label to the first and second panels that they viewed. Compared to round three, respondents responded 7.6% more to round one and 2.4% more to round two labels. There are no differences across the later rounds.

This would primarily be an issue if the survey is not balanced between rounds one and two and rounds three and seven. For example, if there were a large number of respondents who received the government office building treatment in the first round, and the first round is over-weighted generally, our inferences may be subject to bias. However, this is not the case – the randomization has the same distribution over treatments for the two sets of rounds. Figures S13 and S14 contain the distribution of treatments across the first and second round against the third through seventh rounds. There are no pronounced differences in the treatment distributions.

Finally, we include the AMCE values calculated by round in Figure S15. There are no clear trends in AMCE values between the first rounds and the later rounds. There are only five cases where the 95% confidence interval for an AMCE within a round does not contain the mean overall AMCE for all rounds. That is Method/virus and Method/phishing in the first round, Agent/unknown in the fourth round, and Target/government office and Outcome/theft of private information for the seventh round.

Table S9: Response by Round

Dependent Variable:	TerrorBinary
Model:	(1)
Variables	
Round 1	0.0761***
	(0.0102)
Round 2	0.0244**
	(0.0106)
Round 4	-0.0119
	(0.0108)
Round 5	-0.0023
	(0.0106)
Round 6	-0.0069
	(0.0106)
Round 7	-0.0007
	(0.0104)
Fixed-effects	
ID	Yes
Fit statistics	
Observations	21,252
R^2	0.23079
Within R^2	0.00575

Clustered (ID)) standard-errors in parentheses
*Signif. Codes: ***: 0.01, **: 0.05, *: 0.1*

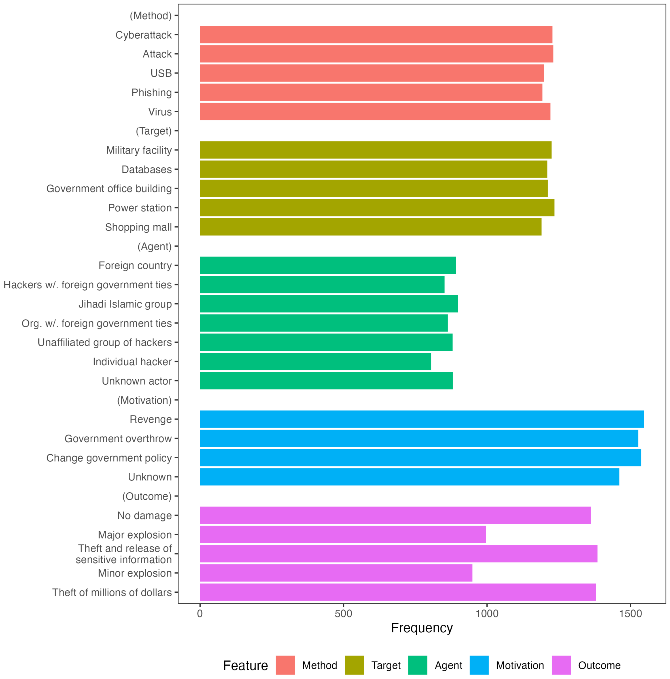

Figure S13: Rounds 1-2

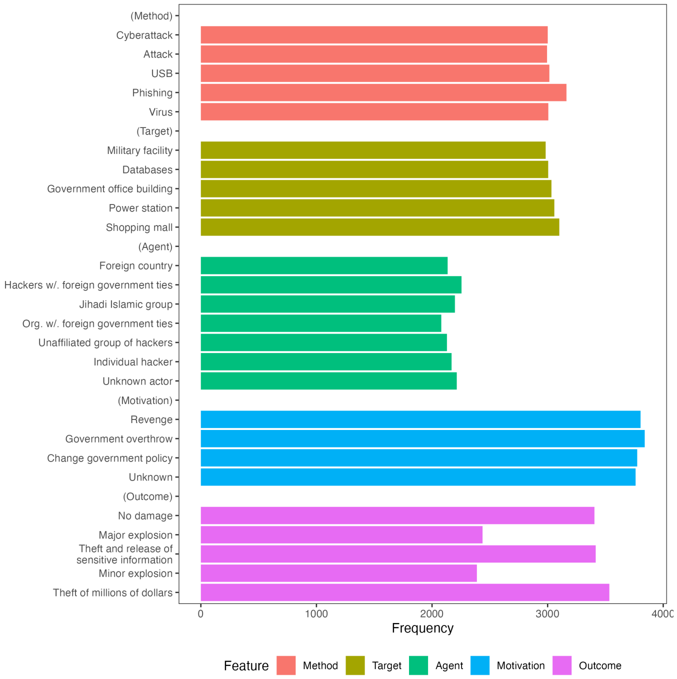

Figure S14: Rounds 3-7

Figure S15: AMCEs by Round

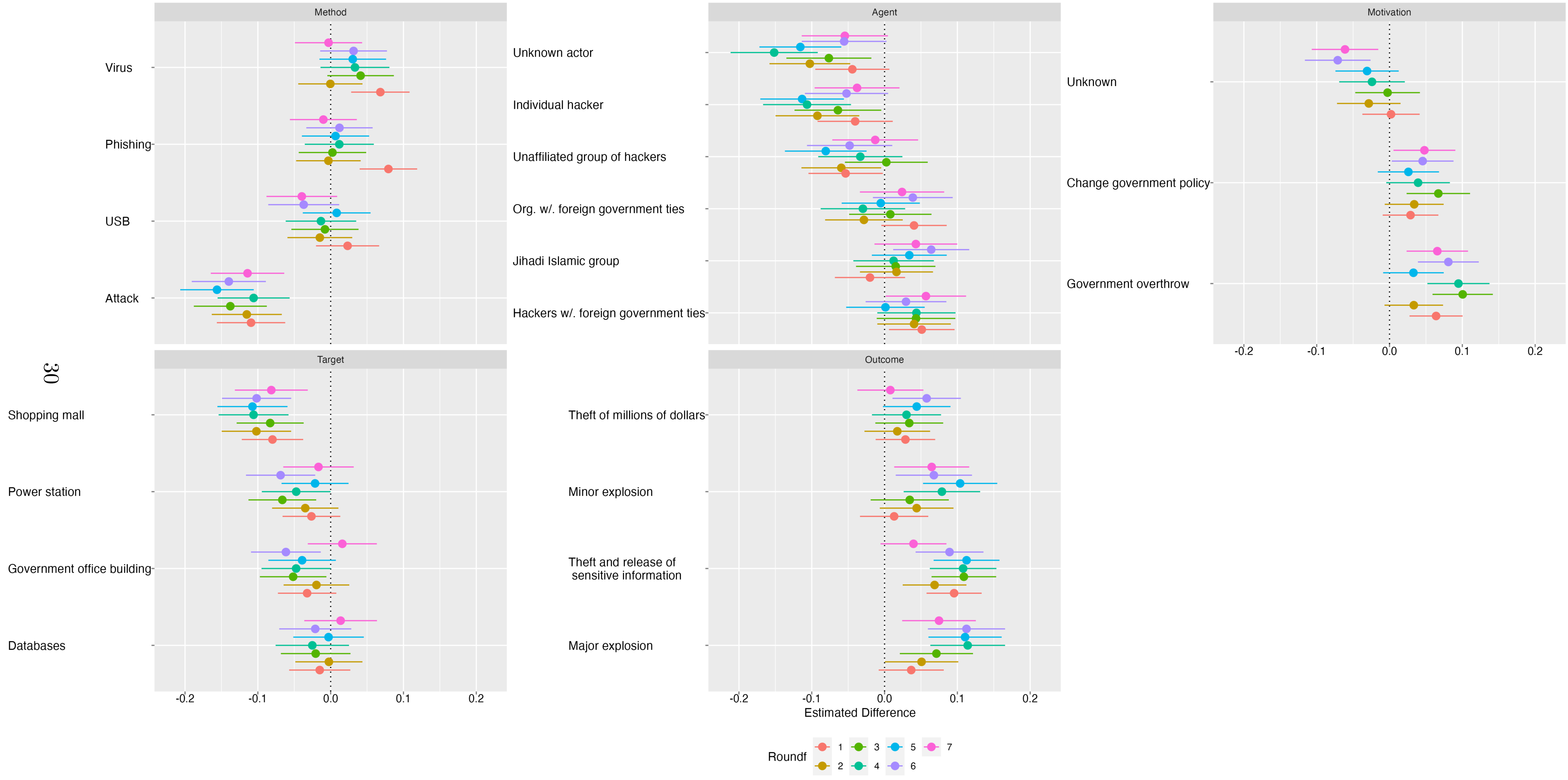

6 References

Berinsky, A. J., G. A. Huber, and G. S. Lenz (2012). Evaluating online labor markets for experimental research: Amazon. com's mechanical turk. *Political analysis 20*(3), 351–368.

Canetti, D., J. Gubler, and T. Zeitzoff (2021). Motives don't matter? motive attribution and counterterrorism policy. *Political Psychology 42*(3), 483–499.

Greszki, R., M. Meyer, and H. Schoen (2015). Exploring the effects of removing "too fast" responses and respondents from web surveys. *Public Opinion Quarterly 79*(2), 471–503.

Hainmueller, J., D. J. Hopkins, and T. Yamamoto (2014). Causal inference in conjoint analysis: Understanding multidimensional choices via stated preference experiments. *Political analysis 22*(1), 1–30.

Hauser, D. J. and N. Schwarz (2016). Attentive turkers: Mturk participants perform better on online attention checks than do subject pool participants. *Behavior research methods 48*(1), 400–407.

Kostyuk, N. and C. Wayne (2020). The microfoundations of state cybersecurity: Cyber risk perceptions and the mass public. *Journal of Global Security Studies*.

Leeper, T. J., S. B. Hobolt, and J. Tilley (2020). Measuring subgroup preferences in conjoint experiments. *Political Analysis 28*(2), 207–221.

Liu, M. and L. Wronski (2018). Examining completion rates in web surveys via over 25,000 real-world surveys. *Social Science Computer Review 36*(1), 116–124.

Palan, S. and C. Schitter (2018). Prolific. ac—a subject pool for online experiments. *Journal of Behavioral and Experimental Finance 17*, 22–27.

Sánchez, R. P. and C. Y. García (2016). Mortality salience, political orientation and minimization of terrorists' secondary emotions. *Psicothema 28*(1), 47–52.
